# Amplified centrosomes and mitotic index display poor concordance between patient tumors and cultured cancer cells

**DOI:** 10.1038/srep43984

**Published:** 2017-03-08

**Authors:** Karuna Mittal, Da Hoon Choi, Angela Ogden, Shashi Donthamsetty, Brian D. Melton, Meenakshi. V. Gupta, Vaishali Pannu, Guilherme Cantuaria, Sooryanarayana Varambally, Michelle D. Reid, Kristin Jonsdottir, Emiel A. M. Janssen, Mohammad A. Aleskandarany, Ian O. Ellis, Emad A. Rakha, Padmashree C. G. Rida, Ritu Aneja

**Affiliations:** 1 Department of Biology, Georgia State University, Atlanta, GA, USA; 2 Department of Pathology, West Georgia Hospital, Lagrange, GA, USA; 3 Department of Gynecologic Oncology, Northside Hospital Cancer Institute, Atlanta, GA, USA; 4 Department of Pathology, University of Alabama, Birmingham, AL, USA; 5 Department of Pathology, Emory University School of Medicine, Atlanta, GA, USA; 6 Department of Pathology, Stavanger University Hospital, Stavanger, Norway, USA; 7 Division of Cancer and Stem Cells, School of Medicine, University of Nottingham and Nottingham University Hospitals NHS Trust, City Hospital Campus, Nottingham, NG5 1PB, UK; 8 Novazoi Theranostics, Inc., Rolling Hills Estates, CA, USA.

## Abstract

Centrosome aberrations (CA) and abnormal mitoses are considered beacons of malignancy. Cancer cell doubling times in patient tumors are longer than in cultures, but differences in CA between tumors and cultured cells are uncharacterized. We compare mitoses and CA in patient tumors, xenografts, and tumor cell lines. We find that mitoses are rare in patient tumors compared with xenografts and cell lines. Contrastingly, CA is more extensive in patient tumors and xenografts (~35–50% cells) than cell lines (~5–15%), although CA declines in patient-derived tumor cells over time. Intratumoral hypoxia may explain elevated CA *in vivo* because exposure of cultured cells to hypoxia or mimicking hypoxia pharmacologically or genetically increases CA, and HIF-1α and hypoxic gene signature expression correlate with CA and centrosomal gene signature expression in breast tumors. These results highlight the importance of utilizing low-passage-number patient-derived cell lines in studying CA to more faithfully recapitulate *in vivo* cellular phenotypes.

Cancer has always been reckoned as a mass of abnormal cells growing rapidly in a deregulated manner. This basic rationale underlies the inception of chemotherapeutic strategies targeting mitosis and development of antimitotic drugs. Since cancer cells divide at a more rapid rate than normal cells, disruption of mitosis has been perceived as the most effective and selective therapeutic strategy against malignant cells. Although mitosis-targeting drugs, such as inhibitors of Aurora kinases, Polo-like kinases, and Kinesin-spindle protein, have been very successful in preclinical trials, their poor performance in the clinical setting has raised doubts about the relevance of this chemotherapeutic strategy^1^. Multiple studies affirm that the rationale undergirding the development of mitosis-targeting drugs is flawed as, frequent mitosis is not a hallmark of human cancers, as previously postulated^1^,^2^. In contrast with immortalized cell cultures and xenograft models, which are most frequently used to assess the efficacy of antimitotic drugs, human tumors tend to have very low mitotic rates (with the mean mitotic index in many tumor types being <1%)^2^. Furthermore, the mean doubling time of a variety of human tumors is >100 days, much higher than that of tumors in pre-clinical models. Although recent cell culture studies have been highly informative, they bear limited conformity with events *in vivo*. Another important factor affecting the potential relevance of cell culture data is drug bioavailability. The drug concentration in the tumor microenvironment varies significantly *in vivo*, with drug concentrations rising, peaking, and falling as the drug circulates and then is removed from the body. In a study evaluating single-cell responses to the antimitotic drug paclitaxel in murine xenograft tumors as compared with cell culture, mitotic frequency was found to be lower in tumors than in cell culture^3^. Interestingly, the peak mitotic index in tumors exposed to paclitaxel was lower and the tumor cells survived longer after mitotic arrest, becoming multinucleated rather than dying directly from mitotic arrest, as opposed to cell cultures. Thus, the *in vivo* tumor microenvironment was found to be far less pro-apoptotic than the environment of cultured cells.

Another cancer cell-specific trait, CA, which refers to the presence of supernumerary or abnormally large centrosomes^4^,^5^, occurs early in pre-cancerous and pre-invasive lesions and is linked to aggressiveness in several types of cancer. CA is believed to drive tumor progression by promoting chromosomal instability and the generation of aggressive tumor clones that are more capable of rapid metastasis. However, the presence of more than two centrosomes within a cell may result in the formation of multipolar spindles, leading to “mitotic catastrophe”^4^ and eventual cell death. To avoid this, cancer cells cluster supernumerary centrosomes into two polar groups to allow formation of a “pseudobipolar” mitotic spindle, a phenomenon that leads to their ultimate survival^4^,^5^,^6^,^7^,^8^. Given that cancer cells rely heavily on centrosome clustering mechanisms for viability, putative centrosome declustering agents have emerged as promising anticancer drugs^9^,^10^,^11^,^12^. Most studies of these drugs rely on cancer cell lines and tumor cell line xenograft models, yet it is unknown how faithfully they recapitulate the profound CA often observed in patient tumors or whether there is a potentially superior model.

Herein, we quantified the prevalence of mitoses and CA in patient tumors compared with tumor cell lines and tumor cell line xenografts. We report that CA, but not mitotic index, is found at high levels in patient tumors, suggesting that CA may be a more valuable chemotherapeutic target than mitosis. We also found that CA progressively declines and mitotic index progressively increases in culture, suggesting differences exist between the *in vivo* and *in vitro* microenvironments that have important ramifications for experimental design. Most often, cells cultured *in vitro* are supplied with copious amounts of oxygen, perhaps to fulfill the metabolic requirement of the voraciously growing log-phase cancer cells. However, in solid tumors, the oxygen concentrations in many regions of the tumor may be severely inadequate resulting in a hypoxic tumor deprived of oxygen^13^. We report here that induction of hypoxia or mimicking hypoxic conditions induces CA *in vitro* via HIF-1α. Moreover, HIF-1α expression was found to correlate with CA in breast tumors. Ultimately, our study emphasizes the limitations of traditional cell culture models for studying CA and highlights the importance of low-passage patient-derived cell lines as being more representative of the true clinical scenario.

## Results

### Mitotic index is lower in patient tumors than tumor cell lines and xenografts

**Figure 1:**
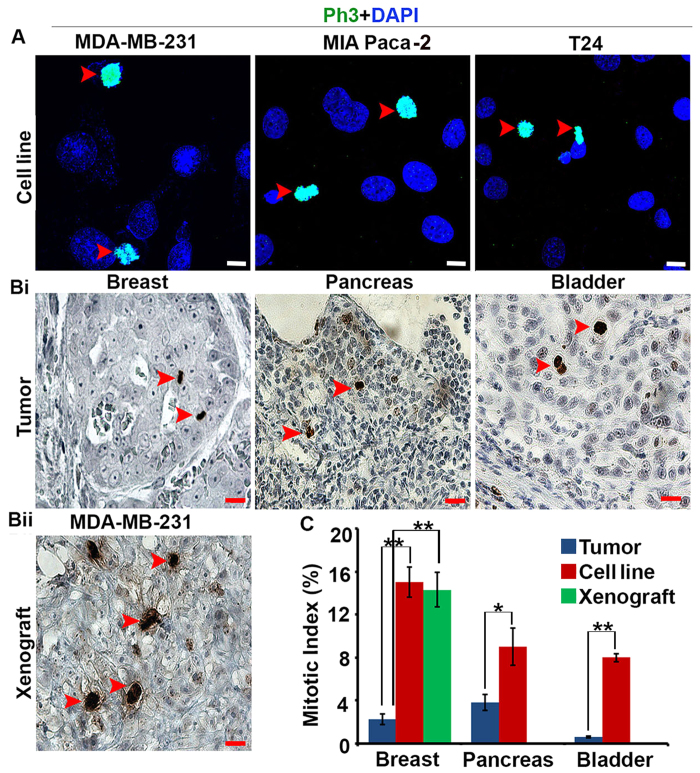
Human tumors display lower mitotic indices than tumor cell lines and xenografts. (**A**) Representative immunofluorescent confocal micrographs of tumor cell lines labeled for the mitotic marker phosphohistone H3 (Ph3). Red arrows: Ph3-positive cells. Scale bar (white), 5 μm. (**Bi,Bii**) Representative immunohistochemical micrographs of a patient breast tumor and an MDA-MB-231 xenograft labeled for Ph3. Red arrows: Ph3-positive cells. Scale bar (red), 20 μm (**C**) Mitotic indices in patient tumors, tumor cell lines, and MDA-MB-231 xenografts. *p < 0.05, **p < 0.01.

To corroborate the view that tumor cells in patients’ bodies are not as mitotically active as cancer cells *in vitro*, we first compared the mitotic indices of patient tumors and established tumor cell lines from different tissue types, including breast, pancreas and bladder. To this end, we quantitated phosphohistone H3 (Ph3)-positive (i.e., mitotic) cells microscopically in 20 paraffin-embedded patient tumor samples (surgical resection) for each cancer type as well as their representative established tumor cell lines. In addition, breast tumor cell line xenografts were analyzed. Descriptive statistics regarding patient and clinicopathologic characteristics for tumor samples utilized are given in Tables 1, 2 and 3. Mitotic indices were approximately 7-fold, 3-fold, and 8-fold higher in MDA-MB-231 breast cancer cells, MIA PaCa-2 pancreatic cancer cells, and T24 bladder cancer cells compared with patient tumors of the respective cancer types. By contrast, mitotic indices were similar between MDA-MB-231 xenografts and cell cultures (Fig. 1A,B). These studies suggest that established tumor cell lines, whether in cultures or xenografted in nude mice, display higher mitotic indices than patient tumors.

### CA is higher in patient tumors and xenografts than tumor cell lines

Having established the low frequency of mitoses in various patient tumor types, we next compared the extent of CA between patient tumor samples and respective tumor cell lines for each cancer type. Breast tumor cell line xenografts were also analyzed. To accomplish this, we microscopically visualized centrosomes in 20 cases for each tissue type along with the specific cell lines. Centrosomes were immunofluorescently stained with anti γ tubulin antibody and co-stained with DAPI. Basically, CA can be of two types: numerical and structural. Numerical aberration can arise from several processes but the main mechanism underlying this phenotype is deregulation of the centrosome duplication cycle, which leads to centriole overduplication and formation of supernumerary centrosomes. Another cause of numerical amplification is failure of cytokinesis, owing to which polyploid cells with supernumerary centrosomes are generated. On occasion, numerical amplification arises from fragmentation of the pericentriolar matrix (PCM)^14^. Similarly, several factors account for structural defects which includes accumulation of excessive PCM around the centrioles (likely due to deregulated expression of genes coding for centrosomal components or altered posttranslational modifications), resulting in centrosomes that appear altered in size^15^. Another possible reason for structural aberration can be tight clustering of centrosomes, which thus cannot be individually distinguished. Third possible reason for this can be structural defects in centrioles; this is a completely unexplored area because the size of normal centriole is very small and requires very sophisticated microscopy techniques especially for tumor samples^4^.

**Figure 2:**
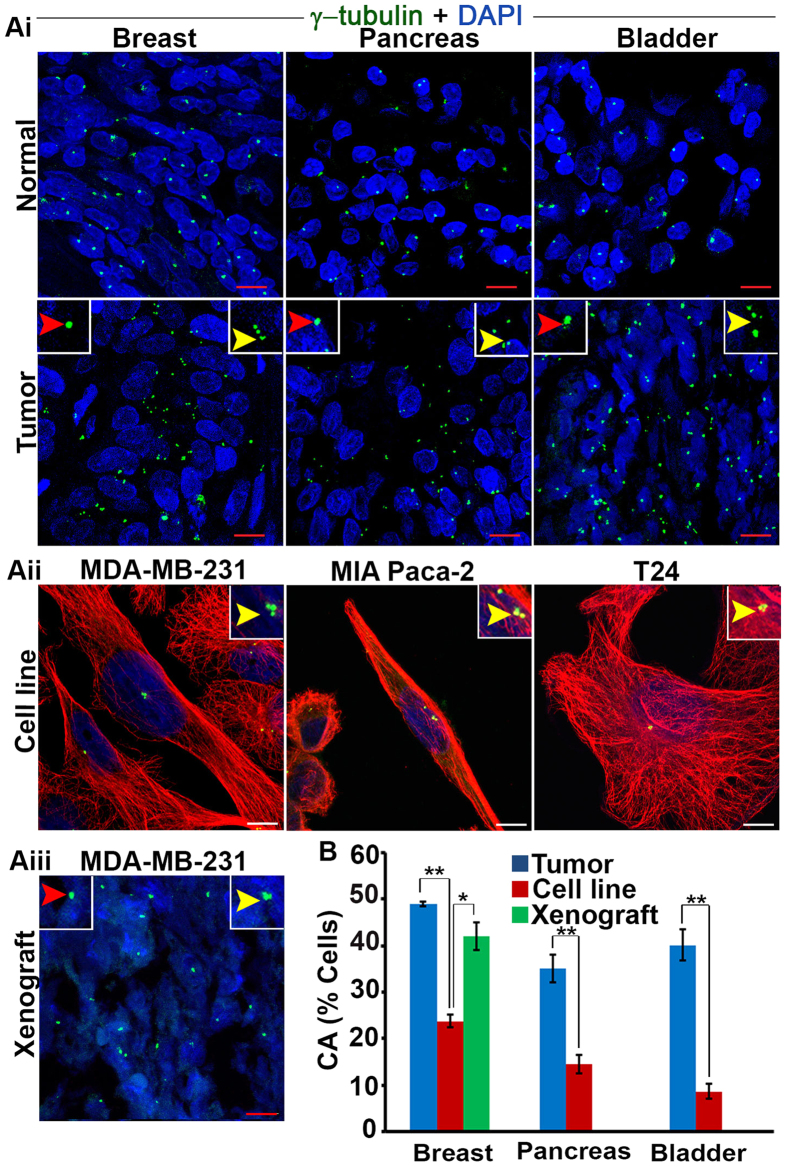
Human tumors have high centrosome aberrations compared to cultured cells. (**Ai**) Bladder, pancreatic, and breast tumors along with normal adjacent tissue immunostained for centrosomes (γ-tubulin, green) and counterstained with DAPI (blue). Yellow arrows, numerical centrosome aberration; red arrows, structural centrosome aberration. Scale bar (red), 20 μm. (**Aii**) Confocal micrographs of centrosome aberrations and clustering in various tumor cell lines. Centrosomes and microtubules were immunolabeled for γ-tubulin (green) and α-tubulin (red), respectively, and DNA was counterstained with DAPI (blue). Scale bar (white) 5 μm. (**Aiii**) Confocal micrographs of centrosome aberrations and clustering in MDA-MB-231 xenografts. Scale bar (red), 20 μm. (**B**) Quantitation of centrosome aberrations in human tumors, tumor cell lines, and tumor cell line xenografts. 500 cells were counted in each case. *p < 0.05, **p < 0.01.

In light of the numerous challenges mentioned above, we used the volumes of the γ-tubulin foci as indicators of structural centrosome aberration. While pancreatic, bladder, and breast tumors exhibited 35%, 36%, and 50% CA, respectively, their corresponding cultured cell lines exhibited 15%, 10% and 23% CA, respectively (Fig. 2A,B). Centrosome clustering was extensive among patient tumors with CA (see inset, Fig. 2Ai). Next, we determined whether CA in the MDA-MB-231 cell lines persists following subcutaneous implantation into nude mice. Remarkably, CA in tumor xenografts excised at the end of six weeks was nearly double that of the native MDA-MB-231 cell line, similar to the level of CA found in patient breast tumors (Fig. 2B). Taken together, these studies clearly demonstrate a high prevalence of CA in human tumors and MDA-MB-231 xenografts but not in cultured tumor cell lines and suggest that differences between the *in vivo* tumor microenvironment and culture plate are at least partly responsible for this observation.

### CA and mitotic index in patient-derived tumor cells change differently with passaging

**Figure 3:**
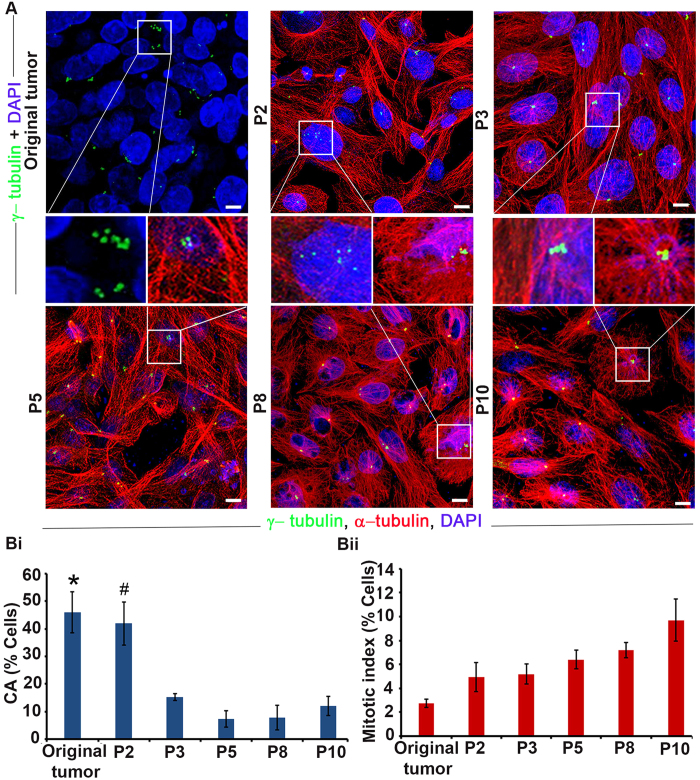
Centrosome aberrations and mitotic index in patient tumors and patient-derived tumor cells with passaging. (**A**) Confocal micrographs of centrosome aberrations and mitotic figures in the original patient tumor and cells isolated from the tumor and cultured through passage 10 (P10). Insets: centrosome aberrations and clustering. Scale bar, 5 μm. (**Bi**) Quantitation of centrosome aberrations at various passage numbers compared with the original tumor. * and ^#^indicate that CA is significantly higher in the original tumor and P2, respectively, when compared with P3, P5, P8 and P10 (p < 0.05). (**Bii**) Mitotic index at various passage numbers compared with the original tumor.

Our observations of the vast disparity in the degree of CA observed in patient tumors and cultured tumor cell lines cast doubt on the clinical relevance of tumor cell lines that are extensively utilized for studying CA. We thus reasoned that patient-derived tumor cell lines at a low passage number may mimic the cellular traits observed in tumor tissues and can emerge as a more useful representative model to conduct *in vitro* studies. We thus examined the degree of CA in patient-derived tumor cell lines by isolating tumor cells from a triple-negative breast cancer (TNBC) and quantitating CA with passaging. CA in the original tumor sample was ~45% (Fig. 3A,Bi). Intriguingly, when tumor cells were dissociated from the tumor mass and cultured, CA progressively declined after passage 2. Passage 3 cells showed a significant 3-fold reduction in CA compared with passage 2 cells (Fig. 3Bi), and by passage 5 the degree of CA fell to ~10%, a level that and was sustained through passage 10 (Fig. 3A,B). We also observed extensive centrosome clustering in cells from passages 2 and 3 as well as in the original tumor tissue (Fig. 3A, inset). Taken together, our data underscore the higher concordance of centrosomal traits between low passage number (passage 2–3) patient-derived tumor cells and cells found in patient tumor tissues and xenografts.

In addition, we assessed the change in mitotic index in patient-derived tumor cells with passaging, which differed remarkably from our observations of CA with passaging. The mitotic index did not change significantly until passage 10, at which time it was ~3-fold higher than in the original tumor (Fig. 3Bii). Taken together, these experiments reveal striking differences in the pace and direction of changes in CA and mitotic index from intratumoral values in patient-derived tumor cells in culture, suggesting that centrosome homeostasis and mitosis are differentially impacted by differences in the *in vivo* and *in vitro* microenvironments.

### Hypoxia enhances CA via HIF-1α in cultured cells

**Figure 4:**
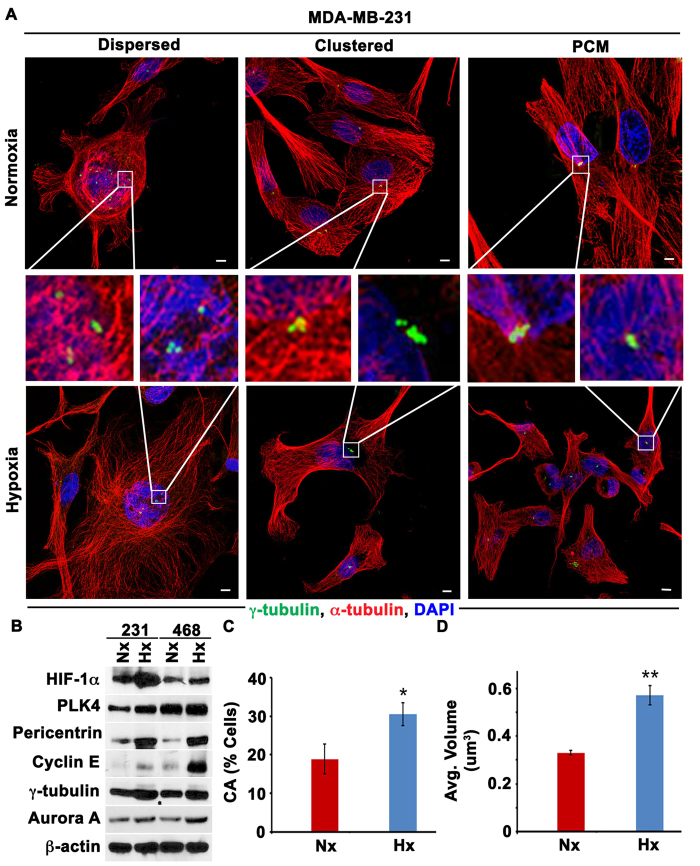
Hypoxia enhances centrosome aberrations. (**A**) Confocal micrographs of centrosome aberration in MDA-MB-231 cells after 48 h of hypoxia (Hx) or normoxia (Nx). Both numerical centrosome aberrations (Supernumerary centrosomes dispersed or clustered) and structural centrosome aberrations (“PCM,” indicating abnormally large individual γ-tubulin foci) were observed. Scale bar, 5 μm. (**B**) Immunoblots of the hypoxia marker HIF-1α, proteins whose overexpression drives centrosome amplification (PLK4, Cyclin **E**, and Aurora **A**), and centrosome structural proteins (pericentrin and γ-tubulin) in MDA-MB-231 cells exposed to 24 and 48 h of hypoxia (Hx). (**C**) Quantitation of centrosome aberrations in MDA-MB-231 48 h after hypoxia. Scale bar, 5 μm. (**D**) Average centrosomal volumes in normoxic (Hx) and hypoxic (Hx) MDA-MB-231 cells. *p < 0.05, **p < 0.001.

Given that a hypoxic microenvironment is one of the major potential differences between tumor cells *in vitro* and *in vivo*, we rationally hypothesized that hypoxia could underlie the divergence in CA observed *in vivo*, both in patient tumors and MDA-MB-231 xenografts (which have been shown to be hypoxic^16^), and established tumor cell lines grown *in vitro*, where oxygen is abundant. To test this hypothesis, we exposed MDA-MB-231 and MDA-MB-468 breast cancer cells to hypoxia for 48 h using a hypoxic chamber flushed with a 1% O_2_ gas mixture. The presence of hypoxia was confirmed by upregulation of HIF-1α (Fig. 4B). As shown in Fig. 4, cells grown in hypoxic conditions for 48 h showed numerical CA, with both clustered and dispersed centrosomes, as well as structural CA, with enlarged γ-tubulin foci (representing individual centrosomes with excessive γ-tubulin accumulation named as PCM accumulation see representative images in Fig. 4A). Following hypoxia, upregulation of proteins whose overexpression drives CA (Cyclin E, Aurora A, and PLK4) and centrosome structural proteins (pericentrin and γ-tubulin) was observed (Fig. 4B, with additional data and description provided in Supplementary Fig. 4B and Supplementary Table 6), along with a significant ~1.5-fold increase in CA (Fig. 4C). Moreover, the average centrosomal volume in cells grown under hypoxic conditions was nearly double the volume in cells grown under normoxic conditions (Fig. 4D). These results suggest that the presence of hypoxia in patients’ tumors could explain, at least in part, the vast differences in CA observed *in vivo* and *in vitro*.

**Figure 5:**
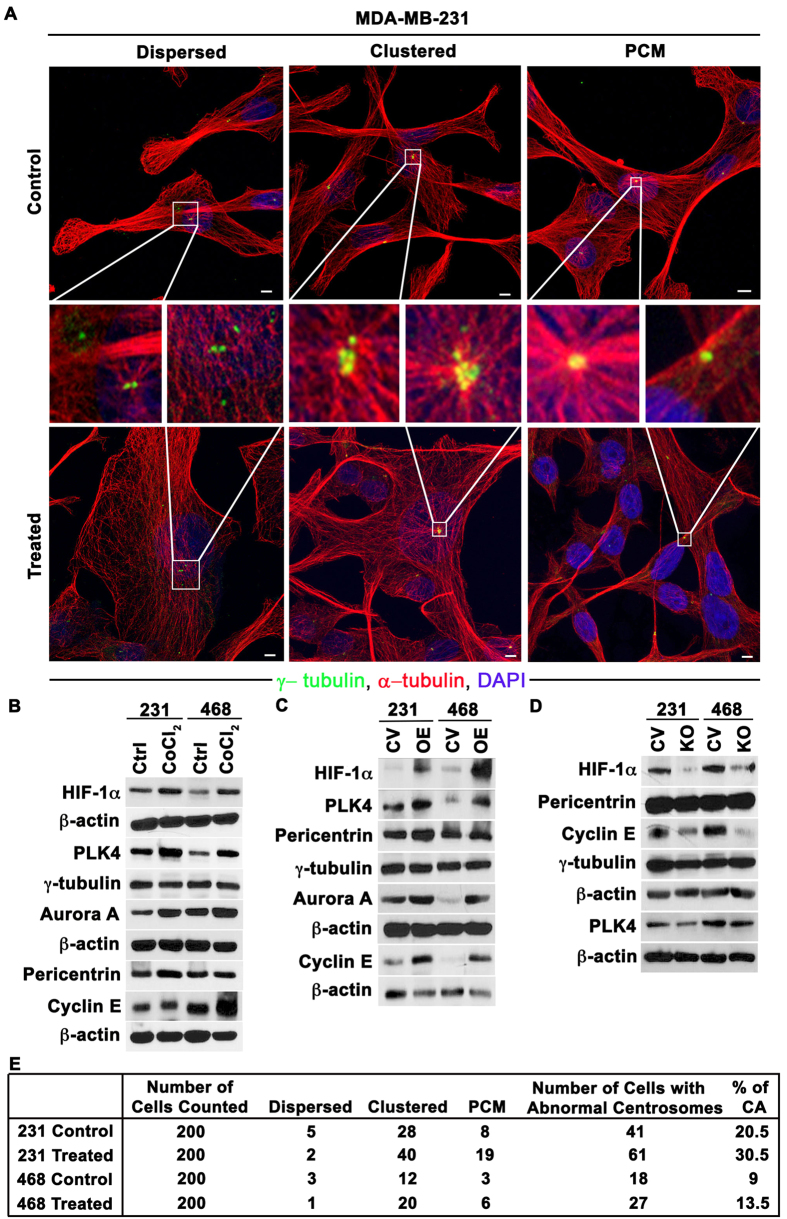
Mimicking hypoxia through pharmacologic and genetic methods enhances centrosome aberrations. (**A**) Confocal micrographs of centrosome aberrations (numerical, including dispersed and clustered configurations, and structural) in MDA-MB-231 cells in control conditions (top panel) and after 24 h CoCl_2_ treatment (bottom panel). Scale bar, 5 μm. (**B**) Immunoblots of HIF-1α and centrosomal proteins in control and CoCl_2_-treated MDA-MB-231 and MDA-MB-468 cells. (**C**) Immunoblots of HIF1α and centrosomal proteins in MDA-MB-231 and MDA-MB-468 transfected with empty vector or degradation-resistant HIF-1α. (**D**) Immunoblots of HIF-1α and centrosomal proteins in MDA-MB-231 and MDA-MB-468 transfected with Cas9-sgRNA (HIF-1α) construct or control vector (pSpCas9-2A-GFP). (**E**) Quantitation of numerical (including dispersed and clustered configurations) and structural (“PCM”) centrosome aberrations per microscopic examination for CoCl_2_ treated and untreated MDA-MB-231 and MDA-MB-468 cells.

To bolster the findings of our hypoxia chamber experiments, we also mimicked hypoxic conditions in normoxia using pharmacologic and genetic methods in MDA-MB-231 and MDA-MB-468 cells. Hypoxia upregulates transcription factor hypoxia inducible factor 1 (HIF-1α) which undergoes proteasomal degradation in normoxic conditions^17^,^18^,^19^. So, to stabilize HIF-1α in normoxic conditions we treated the cells with CoCl_2_, a HIF-1α-stabilizer^20^, which resulted in a ~1.5-fold increase in the CA compared with untreated cells (Fig. 5A,E and Supplementary Fig. 2A), similar to what we found in the hypoxia chamber experiments. To further characterize the observed centrosomal abnormalities, we co-immunolabeled γ-tubulin and centrin-2 (a centriolar marker) and performed the quantitation as described in Supplementary Fig. 3A–C. We found that γ-tubulin foci invariably overlapped with centrin-2 foci in both CoCl_2_-treated and untreated cells, suggesting that the supernumerary γ-tubulin foci observed represent bona fide centrosomes and not mere fragments of pericentriolar material. Moreover, we failed to observe supernumerary centrin-2 foci in enlarged γ-tubulin foci, suggesting that the enlarged γ-tubulin foci represent structurally augmented centrosomes and not supernumerary centrosomes so tightly clustered as to be indistinguishable. The increased CA in CoCl_2_-treated cells was substantiated by protein immunoblotting, which revealed increases in centrosome structural proteins as well as proteins whose overexpression drives centrosome amplification compared with untreated cells (Fig. 5B, with additional data and original blots provided in Supplementary Fig. 4A and Supplementary Table 6). We next treated the cells with dimethyloxalylglycine (DMOG), which stabilizes HIF-1α in normoxic conditions^21^, and MG132, which inhibits 26S proteasomal degradation of HIF-1α^22^. Treatment with either DMOG or MG132 increased CA nearly ~1.5-fold (data shown in Supplementary Fig. 6C), in alignment with our other observations. We confirmed the increase in CA by protein immunoblotting (Supplementary Fig. 6B and Supplementary Table 6).

Next, to confirm that the increase in CA under hypoxia was due to HIF-1α, we overexpressed HIF-1α by transfecting cells cultured under normoxic conditions with GFP-tagged degradation-resistant HIF-1α. Transfected cells showed higher CA (~28%) under normoxic conditions than vector controls (~21%) (Supplementary Fig. 6A) and increase in CA was further confirmed with protein immunoblotting (Fig. 5C). We also knocked-out HIF-1α gene using CRISPR/CAS9 method (details in methods) and exposed these transfected cells to hypoxic conditions and found that levels of centrosomal proteins and proteins whose overexpression drives CA were lower than in vector controls (Fig. 4D and original blots shown in Supplementary Fig. 5 and densitometry values relative to loading control β-actin shown in Supplementary Table 6). In addition, cells transfected with vector control showed higher CA (~21%) (representative images Supplementary Fig. 6A) than HIF-1α knocked out cells, indicating that hypoxia induces CA via HIF-1α. Collectively, these experiments substantiate the paradigm that hypoxic conditions in the tumor microenvironment may account for differences in CA observed between patient tumors/ tumor cell line xenografts and established tumor cell lines.

### Hypoxia is associated with CA in breast tumors

**Figure 6:**
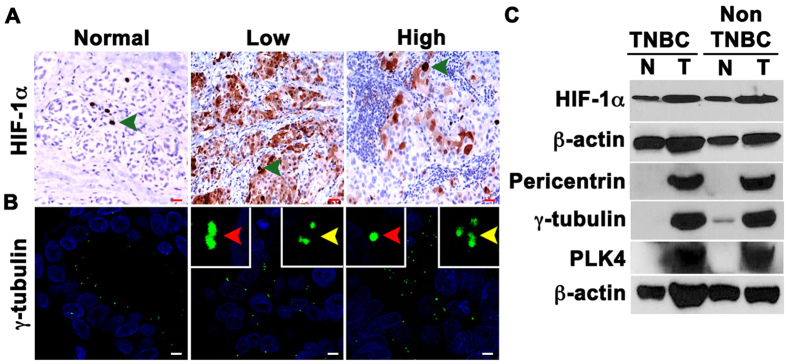
Higher HIF-1α expression is associated with higher CA. (**A**) Representative immunohistochemical micrographs of human breast tumors stained for HIF-1α. Green arrows indicate HIF-1α-positive cells. Scale bar (red), 20 μm. (**B**) Breast tumors along with normal adjacent tissue were immunostained for γ-tubulin (green), and DAPI-stained (blue) to visualize centrosomes, and DNA. (**C**) Immunoblots showing the levels of hypoxia and centrosomal markers in patient tumor samples (T) and their adjacent normal (N) tissues.

We next examined the relationship between HIF-1α levels and CA in breast cancer samples. To this end, we first immunohistochemically labeled 24 breast cancer and uninvolved adjacent normal tissue samples (samples obtained by partial mastectomy pretreatment) for HIF-1α and calculated weighted indices (WIs) for nuclear HIF-1α. Adjacent serial sections from the same tumors were also immunofluorescently labeled for γ-tubulin (Fig. 6A,B). CA was calculated as described in the Supplementary Fig. 1. Descriptive statistics of patient and clinicopathological characteristics, CA levels, and biomarker WIs are given in Table 4. HIF-1α WI was higher in the tumor areas when compared with adjacent normal tissue. In addition, a strong positive correlation between nuclear HIF-1α WI and CA was found in breast tumor samples (Spearman’s rho p = 0.722, p < 0.001). In addition, we found that higher nuclear HIF-1α was associated with worse overall survival (p = 0.041; HR = 1.03). We also compared the expression levels of HIF-1α and centrosome structural proteins (γ-tubulin and pericentrin) in fresh frozen clinical samples and uninvolved adjacent tissue from a pair of patients, one with TNBC and the other with non-TNBC. Immunoblots showed higher expression of HIF-1α and centrosomal proteins in both tumor types in comparison with their normal adjacent tissues (Fig. 6). Finally, using public microarray datasets, we investigated whether centrosomal gene expression is enriched in breast tumors characterized by a hypoxic gene expression signature. We found that breast tumors with high expression of hypoxia-associated genes exhibited higher expression of centrosomal genes than breast tumors with low expression of hypoxia-associated genes regardless of mitotic index (which could otherwise confound analyses given that centrosomal genes are upregulated in mitosis) (Supplementary Fig. 7 and Supplementary Tables 1–5). Furthermore, a score based on the top 10 overexpressed centrosomal genes in breast tumors characterized by high levels of hypoxia-associated genes predicted worse distant metastasis-free survival in 94 node-negative breast cancer patients in multivariable analysis adjusting for various possible confounders (HR = 3.39, p = 0.011), whereas a hypoxia score previously shown to have prognostic ability in multiple cancers^23^,^24^ was non-significant in this full model (see Supplementary text and Supplementary Fig. 7 for more details). Together with our *in vitro* findings, these clinical data analyses support the hypothesis that hypoxia/HIF-1α drive CA in patient breast tumors and contribute to poor outcomes, such as distant metastasis.

## Discussion

While mitosis-targeting drugs have shown remarkable success in immortalized cell lines and tumor xenografts, they have failed to deliver their efficacy in human trials. Our current study provides a rigorous, systematic analysis of the relationship between a universal prognostic factor (mitotic index) and a well-known cancer-cell specific trait and a potential prognosticator (CA) in a spectrum of model systems ranging from cultured cells, preclinical tumor xenografts, patient-derived primary cultures and patient tumors. Our data reconfirm that rapid cell division is not as predominant a trait of human tumors as it is of immortalized cell lines and tumor cell line xenografts. Since preclinical drug development experiments with antimitotic drugs are most often performed using immortalized cell lines or xenograft models, a large fraction of cells in these systems are vulnerable to antimitotic therapy. Therefore, it is not surprising that in human tumors where the fraction of the mitotically active cells is very low, only a small, insignificant fraction of cells are vulnerable to antimitotic drugs. In addition, many studies have shown that the median doubling times for many human tumors are on the order of months or even years, versus only hours or days for immortalized cell lines and tumor xenografts^1^,^25^. The rapid doubling rate of tumors in preclinical models also explain why antimitotic agents prove very effective in these models but fail to show much efficacy against patient tumors. Thus, the lack of response of patient tumors to antimitotic drugs is due to the relative rarity of mitoses and slow doubling rate as highlighted by our study.

While human cancers including colon, breast, bladder, prostate, gliomas, and pancreas show profound CA^26^, we found immortalized cell lines are characterized by a much milder extent of this cell biological trait. The poor concordance between the extent of CA in tumors and cells *in vitro* can thus restrict the utility of cultured cells for studying CA mechanisms *in vitro* as well as for exploring the potential and promise of CA as a therapeutic target or prognostic biomarker. We reason that cancer cells seeking to adapt to and thrive in the tumor microenvironments encounter diverse selection pressures during tumor progression, such as varying levels of oxygen. CA drives chromosomal instability^27^ and thereby generates karyotypic diversity, a trait that is highly desirable for tumors seeking a survival advantage; beyond a certain point however, chromosome instability itself may become a selection pressure that jeopardizes the viability of cancer cells, which may not be able to maintain a chromosomal composition necessary for optimal cell growth. It is likely that in continuous cultures, the diminution of CA is due to cells having achieved a karyotypic composition wherein the persistence of aberrant centrosomes could potentially have deleterious effects. When such a state is attained, CA may itself serve as a selection pressure, thus explaining the attenuation in the extent of aberrations in cultured cells compared to human tumors.

Based on our findings, it seems possible that CA could be a superior target to mitosis, an infrequent event in patient tumors, since a third to half of cells in patient tumors exhibit CA (Fig. 2). Immortalized cell lines, on the other hand, display a much lower degree of CA. This discordance can in part be explained by the presence of hypoxia in the microenvironment of the tumor, which is usually absent *in vitro*. In addition, cells are usually cultured *in vitro* with excessive glucose and growth factors compared with the tumor microenvironment, which helps cultured cells to grow rapidly and thus be more sensitive to antimitotic drugs. Cancer cells dwell within a complex milieu of normal cells, blood vessels, endogenous small molecules, and secreted factors, which together comprise the tumor microenvironment. Hypoxia is one of the hallmarks of the tumor microenvironment, which is critically essential for cancer initiation, progression, metastasis, and drug resistance^28^,^29^. Indeed a major detriment of using cell lines is that the vital interaction of tumor cells with their microenvironment is inherently omitted. Thus, when cancer cells are grown in culture dishes in a two-dimensional plane, the oxygen levels between cells stay relatively equal, which is an improbable setting within a growing three-dimensional tumor in a patient’s body. Moreover, the artificial, non-physiological environment in which cells in laboratory cultures are sustained fails to recapitulate the complex three-dimensional cellular interactions that exist *in vivo*. Another major inadequacy of cell culture is its inability to model the effects of physiologic responses to a tumor, such as the immune response and angiogenesis, two factors known to strongly influence tumor development^30^. Altogether, our study underscores the remarkable disparity in CA and mitosis between patient tumors and model systems, which must be carefully considered when designing experiments to study these phenomena.

Reports indicate that hypoxia, which is known to induce overexpression of HIF-1α, increases Aurora A/STK15 protein levels, which has been well documented to induce CA^31^,^32^,^33^. Our study demonstrates that cells grown under hypoxic conditions exhibit higher CA and Aurora A levels compared with cells grown under normoxic conditions. Many studies have demonstrated that hypoxia is associated with an increased capacity for metastasis^34^,^35^. We^36^ and others^8^,^37^ have shown that supernumerary centrosomes confer cytoskeletal advantages on the cells that harbor them; this could increase directional migration and invasiveness and thus enhance metastatic potential. Although it is possible that hypoxia may favor the proliferation or survival of cancer cells with extra centrosomes and therefore favor the maintenance of CA in the population, our results support the notion that hypoxia induces CA perhaps via promoting overexpression of proteins such as Aurora A. Based on our studies, we speculate that hypoxia may enhance the metastatic potential of cancer cells by inducing CA through upregulation of proteins such as Aurora A, PLK4, and Cyclin E, although a more comprehensive study is needed to investigate this tantalizing research question.

While these preclinical models (both established tumor cell lines and tumor cell line xenografts) are far from ideal, they have been widely used given that the rapid doubling times in such models permit a fast-tracked drug-development timeline. Nonetheless, this perceived advantage rather puts us at a loss when the doubling time itself is in the spotlight and the drug’s activity relies on the preponderance of the mitotic population, which hinges on doubling rate. The brisk doubling times of the preclinical models explain why drugs targeting mitosis proved active in these models but were ineffective against patient tumors^1^,^2^,^25^. Our study highlights the importance of low-passage patient-derived cell line systems as being most representative of the clinical scenario and thus constituting an invaluable experimental model that could better guide drug development and clinical trial design.

Centrosome amplification is now well established as a hallmark of cancer. However, the presence of more than two centrosomes within a cell can be problematic as it may lead to formation of multipolar spindles leading to “mitotic catastrophe”^4^ and cell death. To avoid multipolar spindle formation and subsequent mitotic catastrophe, cancer cells cluster supernumerary centrosomes into two polar groups to allow formation of a “pseudobipolar” mitotic spindle and produce viable daughter cells^6^,^38^. Since our study clearly demonstrates that human tumors display a high frequency of CA, inhibition of centrosome clustering could have afflicted tumor cells to succumb to mitotic catastrophe and be eliminated. Given that cells with CA are suspected to have metastatic potential, antagonizing centrosome clustering could prove to be a strategy to suppress metastasis. Recently, many drugs have been shown to have centrosome declustering, including griseofulvin, noscapine and several of its derivatives (e.g., bromonoscapine and reduced bromonoscapine), the PARP inhibitor PJ-34, and HSET inhibitors like AZ82 and CW069^9^,^10^,^11^,^12^. To discern meaningful activity of these drugs before they are tested in clinical trials as potential centrosome declustering dugs, it is imperative that we consider the shortcomings of our existent cell line models and rather develop robust and relevant preclinical models that mimic cellular traits observed in patient tumors. Our study clearly shows that established tumor cell lines exhibit lower CA than patient tumors and thus may be inferior model systems for testing centrosome targeted drugs than early-passage patient-derived tumor cell lines, which exhibit similar CA to patient tumors.

Undoubtedly, the incongruity in CA between patient tumors and established tumor cell lines depreciates the importance of centrosomes as viable attractive targets and rather overstates mitosis as a target, perhaps resulting in the drug development process being blindsided. Our findings thus underscore the critical need to cautiously identify models that resemble patient tumors more closely in those characteristics/traits that are being targeted and are thus, more clinically relevant. This is the first report to substantiate the previously unrecognized discordance associated with mitotic frequency and the extent of CA between various model systems. Our study emphasizes the limitations of *in vitro* cultures perhaps owing to genomic convergence upon continuous passaging and highlights the importance of low-passage patient-derived cell line system as most representative of the clinical scenario and thus a good preclinical model to study the therapeutic potential of centrosome targeting drugs compared to conventional continuous cell lines. Our study also underscores the significance of CA as a superior chemotherapeutic target. Based upon our findings, we suggest that low-passage patient-derived tumor cells and tumor xenografts could serve as good preclinical models for testing these drugs since the degree of CA found in these models closely resembles that in patient tumors. Taken together, our results suggest that CA could prove to be a better therapeutic target than mitosis owing to its higher incidence in human tumors, which perhaps occurs in low oxygen hypoxic tumor environment.

## Materials and Methods

### Clinical tissue samples

Formalin-fixed paraffin-embedded slides of breast, pancreatic and bladder cancer tissue were procured from Northside Hospital and Emory University Hospital, in Atlanta. The Institutional Review Board of Northside hospital and Emory University approved all aspects of the study. Fresh tumor samples (samples obtained by partial mastectomy pretreatment) were procured from West Georgia Hospital, Lagrange under approved protocols. Methods were carried out in accordance with approved guidelines and informed consent was obtained from all subjects. Descriptive statistics for patient and clinicopathologic characteristics are provided in Tables 1, 2 and 3.

### Established tumor cell lines

MDA-MB-231, MIA PaCa-2 and T24 cell lines were obtained from American Type Cell Culture (ATCC) and were grown in standard conditions. Briefly, grown in Dulbecco’s Modified Eagle’s medium (DMEM) supplemented with 10% Hyclone fetal bovine serum (FBS) and 1% penicillin/streptomycin. Cells were maintained in humidified 5% CO_2_ atmosphere at 37 °C.

### Patient-derived tumor cell lines

Tumor cells were isolated from a TNBC patient tumor (partial mastectomy) obtained from West Georgia hospital. To isolate tumor cells for culture, the tumor tissue was first minced into small pieces and then was digested in a mixture of DMEM/F12 medium containing 2 mg/ml bovine serum albumin, 2 mg/ml collagenase type IV, and 2 mg/ml hyalurodinase at 37 °C for 30–40 min with continuous agitation. After the tumor chunks were completely digested, cells were filtered through a 70 μm mesh, centrifuged at 2,000 rpm for 10 minutes, resuspended in fresh DMEM/F12 supplemented with 10% FBS and 1% penicillin, and plated in 10 mm culture dishes in humidified 5% CO_2_ atmosphere at 37 °C.

### Tumor cell line xenografts

All animal experiments were performed in compliance with Georgia State University (GSU) Institutional Animal Care and Use Committee (IACUC) guidelines. All the animal protocols (including description of experiments and experimenters) were approved by GSU IACUC. For implantation into nude mice, MDA-MB-231 cells were washed with PBS, digested with trypsin, resuspended in DMEM 1X containing 10% FBS, and pooled. After centrifugation, cells were resuspended in Matrigel (BD Biosciences Discovery Labware, Bedford, MA)-DMEM 1X (1:3) at a concentration of 1 × 10^6^ cells/100 μL, 100 μL of which was subcutaneously implanted into the dorsa of 6-week-old female Bald*/nu* mice (Harlan Sprague-Dawley, Indianapolis, IN). Tumor volumes were monitored constantly for 6 weeks, and after that tumors were excised and fixed in 10% formalin, embedded in paraffin, sectioned at 5 μm, and immunolabeled for centrosomes (γ-tubulin) and mitotically-active cells (Ph3).

### Lysate preparation and immunoblotting

Cells were cultured to ~80% confluence and protein lysates were prepared as described previously^39^. Briefly, cells were scraped with 250ul of 1x lysis prepared from 10x cell lysis buffer (Cell Signaling). The 1x lysis buffer contained 1 mM b-glycerophosphate, 20 mM Tris–HCl (pH 7.5), 1 mM Na2EDTA, 1 mM Na3VO4, 150 mM NaCl, 1 mM EGTA, 2.5 mM Na4P2O7, 1 ug/ml leupeptin, and 1% Triton. 10% Protease inhibitor was added to prevent degradation of proteins. Cell lysates were fractionated using 10% SDS-PAGE gel. Fresh tissue sections were sonicated and lysates were then prepared using the same lysis buffer. Polyacrylamide gel electrophoresis was used to resolve the proteins, which were transferred onto polyvinylidene fluoride membranes (Millipore). The Pierce ECL chemiluminescence detection kit (Thermo Scientific) was used to visualize the immune-reactive bands. β-actin was used as loading control. Antibodies used in immunoblot assay are listed in Table 5.

### Immunohistofluoresence staining

Formalin-fixed paraffin-embedded tissue slides were deparaffinized followed by serial rehydration in ethanol baths (100%, 95%, 70% and 50%). Antigens were retrieved by heating in a pressure cooker in citrate buffer (pH 6.0) at psi 15 for 30 min. Blocking was performed by incubating the slides with the ultra-vision protein block (Life Sciences) for 30 min. Tissue samples were then incubated overnight with primary mouse antibody against γ-tubulin (Table 5) at 1:1000 dilution) at 4 °C, followed by washing 3X with PBS. The samples were then incubated with secondary antibody (Alexa-488 anti-mouse) at 1:2000 dilution for 2 h, at 37 °C followed by washing 3X with PBS. Finally, coverslips were mounted with Prolong-Gold Antifade Reagent with DAPI (Invitrogen).

### Immunohistochemical staining and scoring

Deparaffinization and antigen retrieval were performed as described as for immunohistofluoresence staining. Thereafter, the tissues were immunolabeled using antibody against Ph3 (dilution 1:1000) or HIF-1α (1:1000). Ph3-positive cells were counted in 10 randomly selected fields (~500 cells) to determine the percentage of mitotic cells. Enzymatic antibody detection was performed with the Universal LSAB + Kit/HRP (DAKO, CA, USA). HIF-1α staining intensity was scored as 0 = none, 1 = low, 2 = moderate, or 3 = high, and the percentage of positive cells (i.e., with 1+ staining intensity) from 10 randomly selected fields (~500 cells) was determined. The product of the staining intensity and the percent of positive cells (nuclei) constituted the WI.

### Immunocytofluorescence staining

Cells were grown on glass coverslips, fixed with ice-cold methanol for 10 min, and then blocked with 2% bovine serum albumin/1XPBS/0.05% Triton X-100 at 37 °C for 1 h. Coverslips were incubated in primary antibodies against γ-tubulin and α-tubulin at 1:2000 dilution for 1 h at 37 °C, washed with 2% bovine serum albumin/1XPBS for 10 min at room temperature, and then incubated in 1:2000 Alexa 488- or 555-conjugated secondary antibodies (Invitrogen; Carlsbad, CA). Cells were mounted with Prolong Gold Antifade Reagent with DAPI (Invitrogen).

### Microscopy

Images of tissue samples were taken utilizing the Zeiss LSC 700 confocal microscope (Oberkochen, Germany) and were processed with Zen software (Oberkochen, Germany). Magnifications and more details on imaging is provided in individual sections.

### Quantitation of centrosome aberration

Numbers and volumes of γ-tubulin foci were used as indicators of numerical and structural centrosome abberation, respectively. Since γ-tubulin is present in both centrioles and the PCM, above-normal volumes of γ-tubulin foci represent the cumulative structural volume amplification of both PCM and centrioles. Centrosomal volumes were calculated using the 3D measurement module from the Zeiss imaging software. Average centrosomal volumes ranged between 0.22–0.76 μm^3^ in normal breast, 0.20–0.56 μm^3^ in normal pancreas, and 0.20–0.74 μm^3^ in normal bladder tissue. The percentage of cells with >2 centrosomes as quantitated from 10 randomly selected fields (around 500 cells) in tumor areas pre-marked by a pathologist was determined for each tissue type as well as cell lines (Supplementary Fig. 1). CA was calculated as a percentage by adding the percent cells harboring more than two centrosomes and the percent cells harboring centrosomes with volume larger than 0.76 μm^3^, 0.56 μm^3^ and 0.74 μm^3^ for breast, pancreatic and bladder tissues respectively. A more detailed description of the quantitation process along with a schematic is given in the Supplementary materials and Supplementary Fig. 1.

### Induction of hypoxia and mimicking hypoxic conditions

Hypoxia chamber: Cells grown on glass coverslips were either placed in a hypoxic modulated incubator chamber (flushed with 1% O_2_ gas mixture at 20 L/min for 7–10 minutes every 3–6 hrs) or a normoxic incubator. After 48 h, cells were trypsinized and lysates were prepared for immunoblotting assays. To pharmacologically induce hypoxia cells were treated with 100 μM of CoCl_2_ for 24 hrs. Further to stabilize HIF-1α in normoxic conditions cells were treated with 1 mM DMOG (SIGMA) for 24 hrs and 5 μM MG132 for 5 hrs. Glass coverslips having cells were fixed with ice cold methanol and staining was performed as described in cell staining section.

### HIF-1α overexpression

HIF-1α was genetically overexpressed by transfecting cells with GFP-tagged degradation resistant HIF-1α. HA-HIF-1α P402A/P564A-pcDNA3 was a generous gift from Dr. Willian Kaelin (Addgene plasmid # 18955)^40^. Cells at a confluency of ~70% were transfected using Lipofectamine 2000 according to the manufacturer’s instructions.

### HIF-1α gene knock out

The gene knockout of HIF-1α was performed using CRISPR/Cas9 method. Where in, guide RNAs to target the human HIF-1α gene was designed using the (http://tools.genome-engineering.org) source. Two individual sgRNAs were designed to target exon 1 of HIF-1α (sgRNA1, 5′CACCGTTTCTTGTCGTTCGCGCCGC3′; sgRNA2, 5′AAACGCGGCGCGAACGACAAGAAAC 3′). sgRNA-encoding oligonucleotides was cloned into pSpCas9–2A-GFP (PX458) (a generous gift from Feng Zhang (Addgene plasmid # 48138) (using standard procedures www.genome-engineering.org)^41^. Transfection of the MDA-MB 231 and MDA-MB 468 cells was performed as described under the section of HIF-1α OE. As a negative control for the transfection efficiency vector pSpCas9-2A-GFP was used. The pSpCas9(BB)-2A-GFP plasmid was GFP tagged hence the sgRNA and Cas9 expressing cells were sorted using FACS. The sorted GFP positive cells were expanded and the knockout in these cells was verified by exposing these cells to hypoxia followed by immunoblotting for HIF-1α.

### Statistical analyses

Unless otherwise stated in the methods and results sections, statistical analyses were performed using two-tailed Student’s t-tests. The criterion for statistical significance for all analyses was p < 0.05. Survival analysis (simple Cox model) was performed using SPSS Statistics version 21(IBM).

## Additional Information

**How to cite this article:** Mittal, K. *et al*. Amplified centrosomes and mitotic index display poor concordance between patient tumors and cultured cancer cells. *Sci. Rep.* **7**, 43984; doi: 10.1038/srep43984 (2017).

**Publisher's note:** Springer Nature remains neutral with regard to jurisdictional claims in published maps and institutional affiliations.

## Supplementary Material

Supplementary Information

## Figures and Tables

**Table 1:** Descriptive statistics for patient and clinicopathologic characteristics in the analysis of centrosome aberrations and mitotic index in breast tumors.

Variable	Level	Number	Percentage
Race	AA	4	20
	EA	16	80
Gender	Male	0	0
	Female	20	100
	1	9	45
Grade	2	5	25
	3	6	30
	I	10	50
Stage	II	7	35
	III	1	5
	IV	2	10
ER/PR Expression	ER-/PR-	8	40
	ER-/PR +	1	5
	ER+/PR+	11	55
CA (%)	Low (<10%)	0	0
	Moderate (10–40%)	7	35
	High (>40%)	13	65
Mitotic Index	<1	8	40
	1~6	12	60

ER: Estrogen Receptor; PR: Progesterone Receptor; CA: Centrosome Aberration; AA: African American; EA: European American.

**Table 2:** Descriptive statistics for patient and clinicopathologic characteristics in the analysis of centrosome aberrations and mitotic index in bladder tumors.

Variable	Level	Number	Percentage
Race	AA	13	65
	EA	7	35
Grade	1	7	35
	2	0	0
	3	13	65
Invasive Status	Non-invasive	7	35
	Invasive	13	65
CA (%)	Low (<10%)	0	0
	Moderate (10–40%)	11	55
	High (>40%)	9	45
Mitotic Index	<1	18	90
	1–6	2	10

CA: Centrosome aberration; AA: African American; EA: European American.

**Table 3:** Descriptive statistics for patient and clinicopathologic characteristics in the analysis of centrosome aberrations and mitotic index in pancreatic tumors.

Variable	Level	Number	Percentage
Race	AA	9	45
	EA	11	55
Gender	Male	11	55
	Female	9	45
Tumor size (cm)	≤2	3	15
	>2	17	85
Grade	Low	10	50
	High	10	50
PNI	Yes	17	85
	No	3	15
LVI	Yes	15	75
	No	5	25
Stage T	1	2	10
	2	2	10
	3	16	80
Stage N	1	15	75
	0	4	20
	Unknown	1	5
Stage M	Yes	19	95
	No	0	0
	Unknown	1	5
LN Positive	≤5	15	75
	>5	5	25
CA (%)	Low (<10%)	0	0
	Moderate (10–40%)	14	70
	High (>40%)	6	30
Mitotic Index (MI)	<1	5	25
	1–6	9	45
	>6	6	30

CA: Centrosome aberration; AA: African American; EA: European American; PNI: Peri-Neural Invasion; LVI: Lympho-Vascular Invasion.

**Table 4:** Descriptive statistics for patient and clinicopathologic characteristics in the analysis of centrosome aberrations and HIF-1α in breast tumors.

Variable	Level	Number	Percentage
Race	EA	14	58%
	AA	10	42%
Grade	1	1	4%
	2	3	13%
	3	20	83%
Stage	I	10	42%
	II	8	33%
	III	2	8%
	IV	1	4%
	N/A	2	8%
ER/PR/HER2 Expression	ER-/PR-/HER2-	24	100%
CA%	Low (<10%)	5	21%
	Moderate (10%-40%)	16	67%
	High (>40%)	3	13%

**Table 5:** List of antibodies used for western blot (WB), immunofluorescence cell and tissue staining (IF) and immunohistochemical (IHC) tissue staining.

	Technique	Company	Observed Band Size (kDa)	Predicted Band Size (kDa)	Dilution
HIF-1α	WB, IHC	Abcam	110~130	93	1:1000^42^,^42^
PLK4	WB	Abcam	~90	109	1:1000^44^
Pericentrin	WB	Abcam	~72	75	1:1000
Cyclin E	WB	Santa Cruz Biotechnology	45~53	53	1:1000^45^
γ-tubulin	WB, IF	Sigma	46~48	48	1:1000^46^
Aurora A	WB	Abcam	~45	46	1:1000^47^,^48^
α-actin	WB	Santa Cruz Biotechnology	43~45	43	1:1000^49^
Centrin-2	IF	EMD Millipore	~18		1:400
α-tubulin	IF	Sigma	~50–55		1:1000
