# Supplementary material for: Amplified centrosomes and mitotic index display poor concordance between patient tumors and cultured cancer cells
Source: Sci Rep. 2017 Mar 8;7:43984. doi: 10.1038/srep43984 (PMC5341055; doi:10.1038/srep43984)
Supplement: Supplementary Information [file srep43984-s1.pdf]

Supplementary Material

Amplified centrosomes and mitotic index display poor concordance between patient tumors and cultured cancer cells

Karuna Mittal[1], Da Hoon Choi[1], Angela Ogden[1], Shashi Donthamsetty[1], Brian D. Melton[1], Meenakshi. V. Gupta[2], Vaishali Pannu[1], Guilherme Cantuaria[3], Sooryanarayana Varambally[4], Michelle D. Reid[5], Kristin Jonsdottir[6], Emiel A. M. Janssen[6], Mohammad A. Aleskandarany[7], Ian O. Ellis[7], Emad A. Rakha[7], Padmashree C. G. Rida[1,8]*, Ritu Aneja[1]*

Detailed Description of Centrosome Aberrations Quantitation

To evaluate the number of centrosomes in tumor samples per cell, we immunostained centrosomes in tissues (normal and tumor for respective cancer types) with anti-γ-tubulin antibody and counterstained nuclei with DAPI. We imaged these tissue slides at low magnification (20x) employing LSM 700 confocal microscope to capture images of 10 fields of view that encompass several nuclei and centrosomes (Supplementary Fig. 1). For each field we selected a region with 30-35 clearly distinguishable nuclei and defined it as region of interest (ROI) by drawing a boundary around the outer edges of outermost nuclei in the ROI. Next we quantified the number and volume of γ-tubulin foci in each ROI at higher magnification (63X objective). In our study, we defined centrosome aberrations as numerical (dispersed and clustered) and structural aberrations (PCM). The "dispersed" configuration refers to cells with widely dispersed supernumerary centrosomes in interphase. On the other hand, "clustered" refers to cells with supernumerary

centrosomes assembled together in interphase (either individually distinguishable or clustered tightly). The third category, termed “PCM” comprised cells with centrosomes whose volumes were above-normal and were represented as only one γ-tubulin spot, not a cluster of γ-tubulin spots. Centrosome aberrations (CA) was calculated as a percentage by adding percent cells harboring more than two γ-tubulin foci and percent cells harboring γ-tubulin foci with volume greater than upper range of mean centrosomal volumes found in respective normal tissues (Supplementary Fig. 1). Since centrosomes pass through a duplication cycle that involves large volume changes, we needed to define a “normal range” for centrosomal volumes using both adjacent uninvolved tissue from cancer patients and normal tissue for disease-free individuals for each cancer type. To determine the normal range, we analyzed volumes of centrosomes (500 centrosomes for each sample) in adjacent uninvolved tissue from cancer patients (20 samples for each cancer type) and in normal tissues (20 normal tissue samples for breast, pancreas and bladder). Normal tissue samples were obtained from Biomax Inc. in the form of commercial tissue microarrays. We evaluated the volume of centrosomes by using the three-dimensional measurement module in the Zeiss imaging software. Mean volumes of centrosomes in normal breast, pancreatic and bladder epithelial cells ranged between 0.22-0.76 μm^3, 0.20-0.56 μm^3, and 0.20-0.74 μm^3, respectively. The various centrosomal phenotypes observed in hypoxic conditions are represented in Supplementary Fig. 2 and 3. The presence of CA was confirmed via immunoblotting for CA-associated proteins as shown in Supplementary Fig. 4.

Supplementary Figure - 1 (Aneja et al)

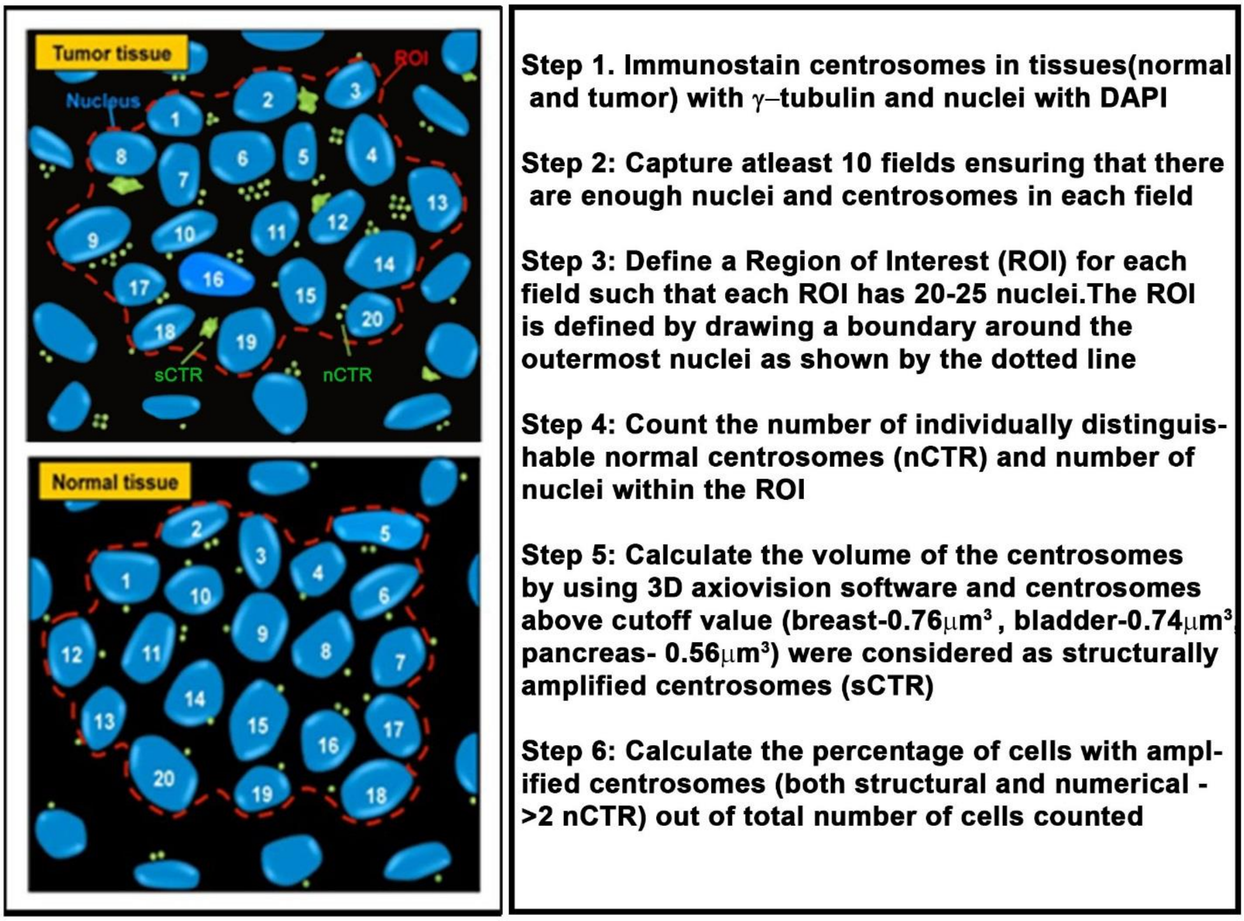

Step 1. Immunostain centrosomes in tissues(normal and tumor) with γ–tubulin and nuclei with DAPI

Step 2: Capture atleast 10 fields ensuring that there are enough nuclei and centrosomes in each field

Step 3: Define a Region of Interest (ROI) for each field such that each ROI has 20-25 nuclei.The ROI is defined by drawing a boundary around the outermost nuclei as shown by the dotted line

Step 4: Count the number of individually distinguis-hable normal centrosomes (nCTR) and number of nuclei within the ROI

Step 5: Calculate the volume of the centrosomes by using 3D axiovision software and centrosomes above cutoff value (breast-0.76μm^3 , bladder-0.74μm^3, pancreas- 0.56μm^3) were considered as structurally amplified centrosomes (sCTR)

Step 6: Calculate the percentage of cells with ampl-ified centrosomes (both structural and numerical - >2 nCTR) out of total number of cells counted

Supplementary Figure 1: Schematic showing the steps followed for quantitation of CA in the cancer tissues, normal tissues and their corresponding cultured cell lines.

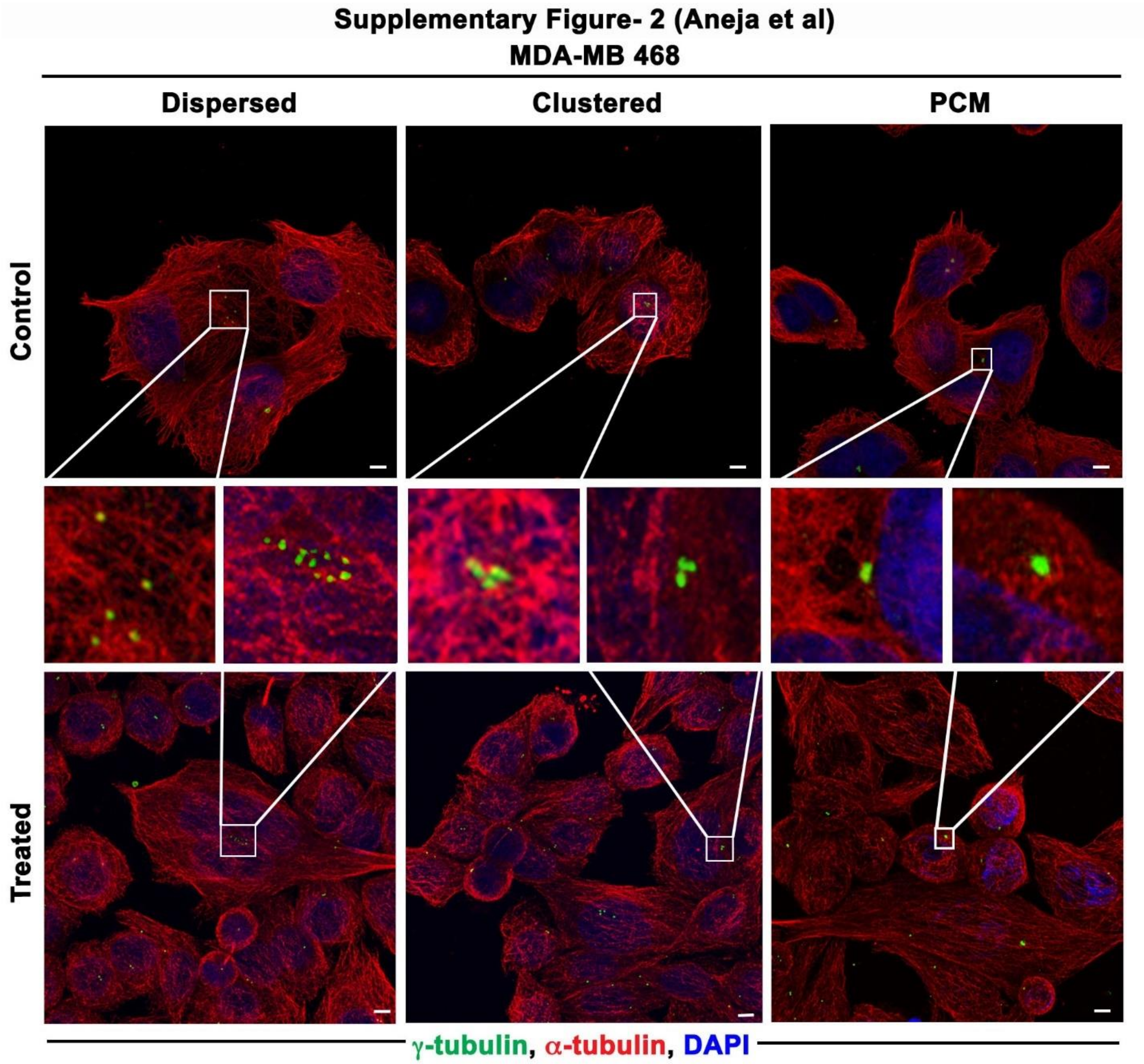

Supplementary Figure 2: Different configurations of Centrosome aberrations observed in MDA-MB-468 after hypoxia induction by treating them with $CoCl_2$**.** Representative immune-micrographs of cells with centrosome aberrations (“dispersed’ refers to cells with centrosomes widely dispersed in interphase; “clustered” refers to cells with supernumerary centrosomes that are individually distinguishable or clustered tightly together in interphase; and “PCM” refers to centrosomes that are abnormally large due to PCM accumulation).

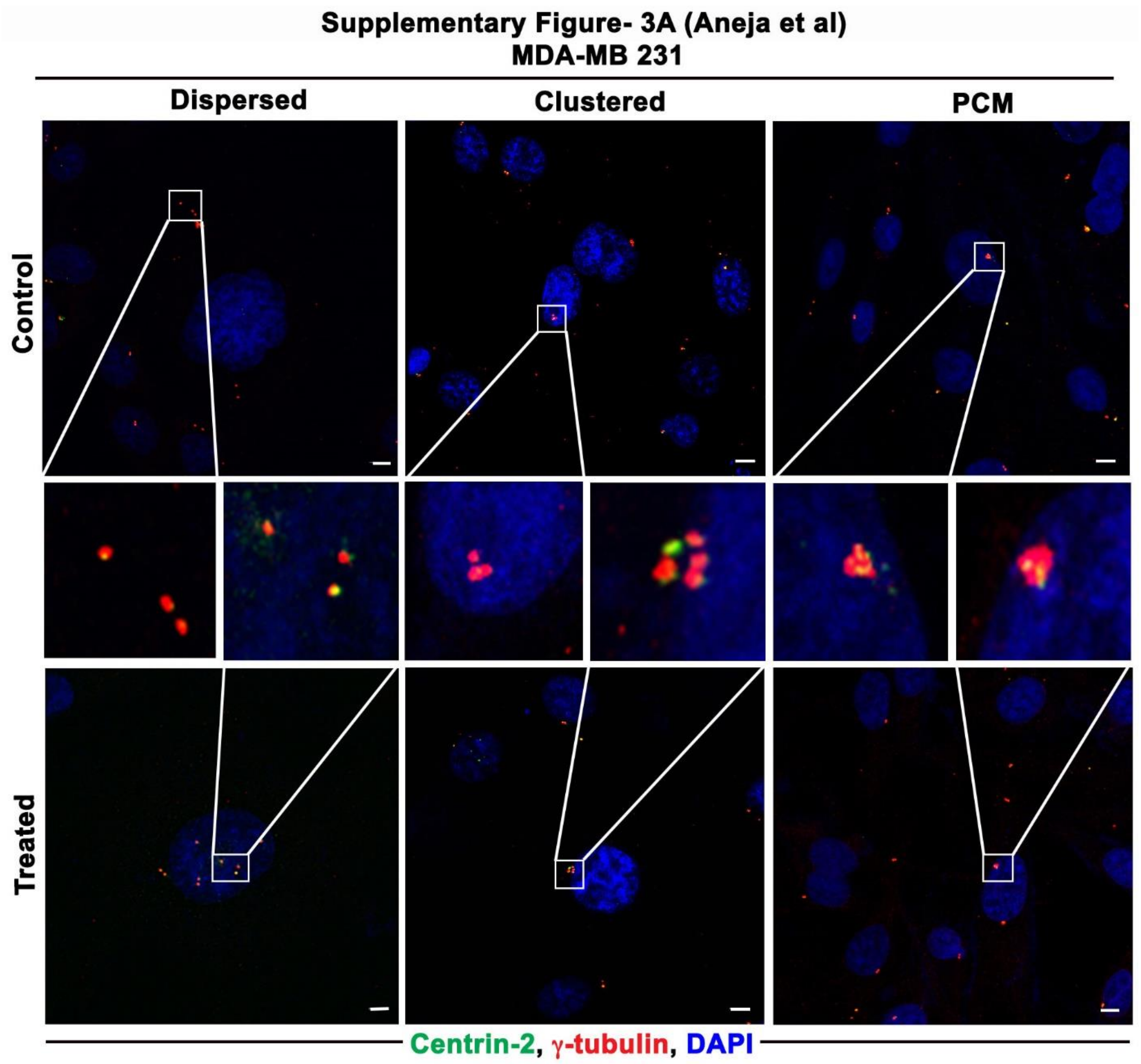

Supplementary Figure 3A: Different configurations of Centrosome aberrations observed in MDA-MB-231 immunostained for centrin-2 (green) and γ-tubulin (red) after treatment with $CoCl_2$. Representative immune-micrographs of cells with centrosome aberrations (“dispersed’ refers to cells with centrosomes widely dispersed in interphase; “clustered” refers to cells with supernumerary centrosomes that are individually distinguishable or clustered tightly together in

interphase; and “PCM” refers to centrosomes that are abnormally large due to PCM accumulation). 200 cells were counted in each condition.

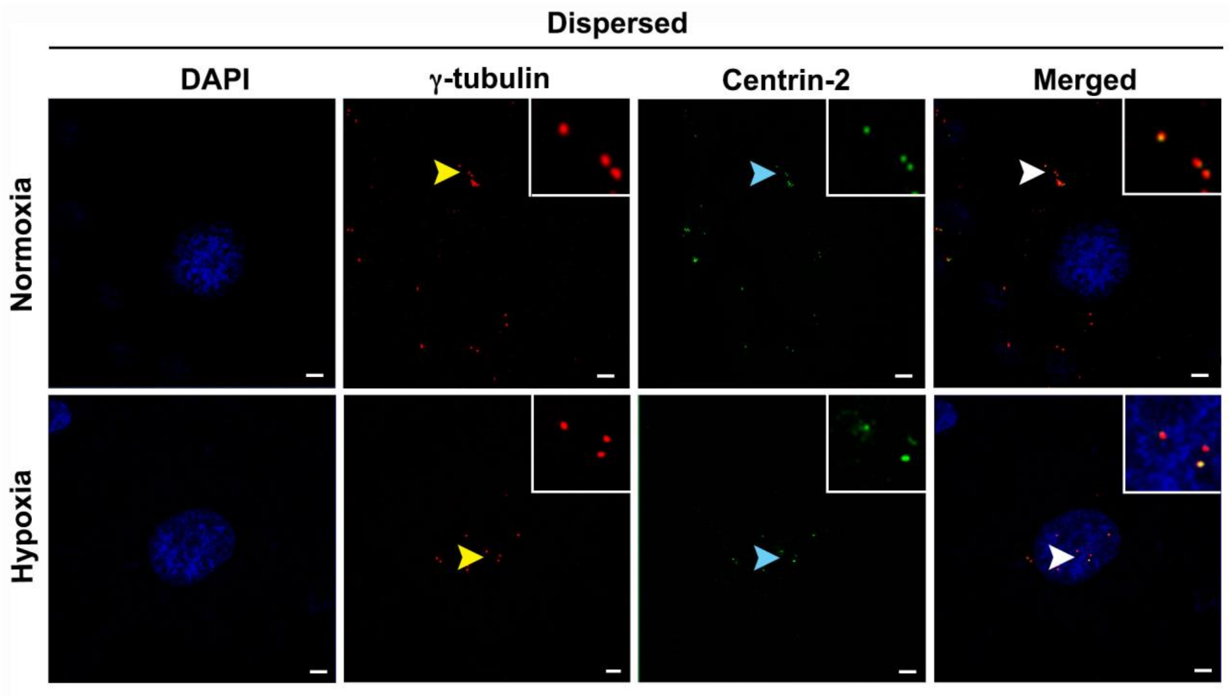

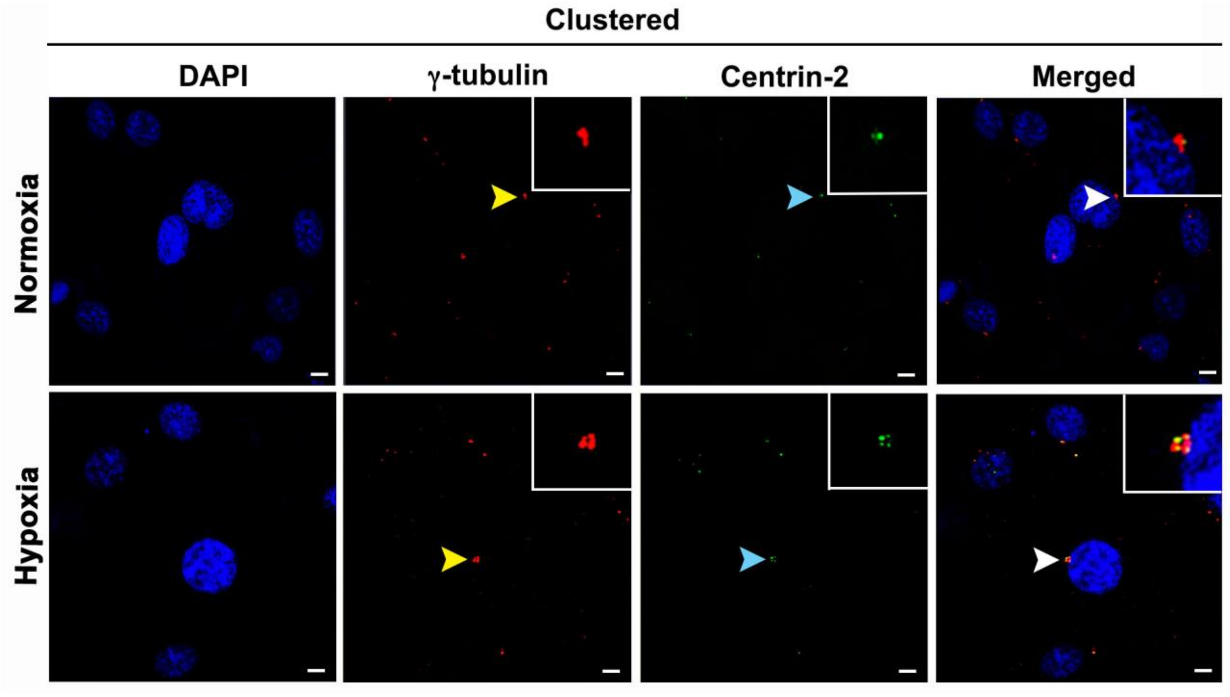

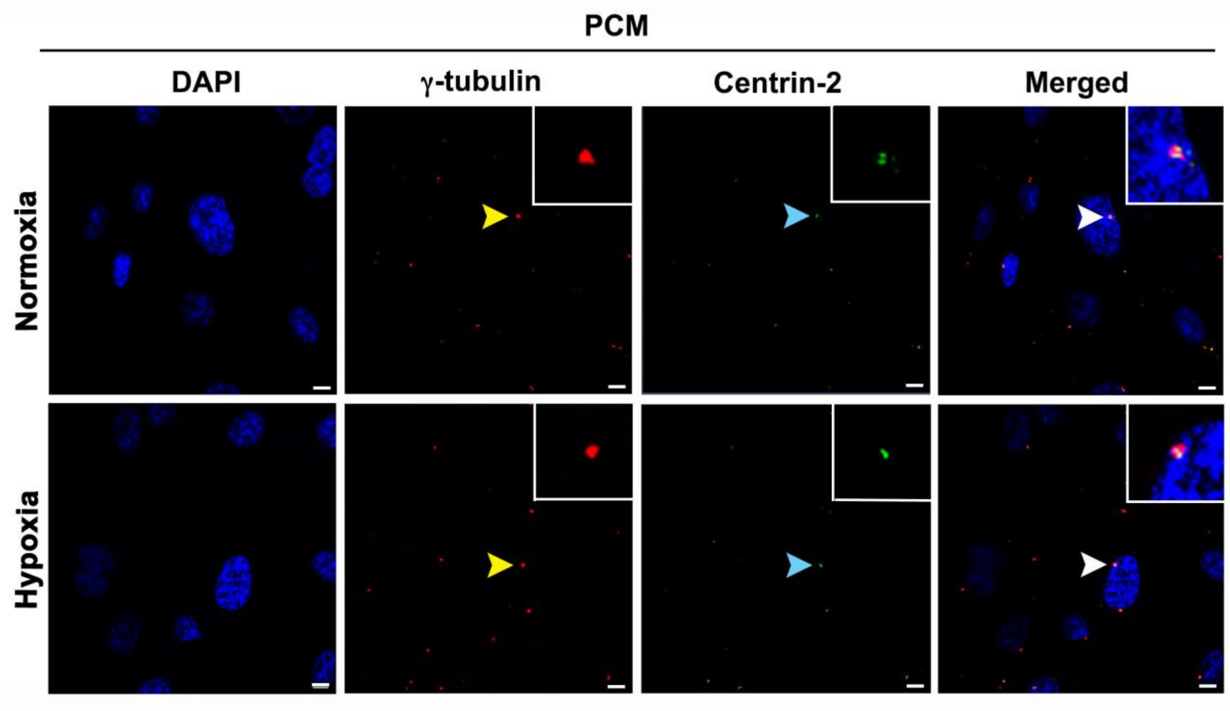

Figure 3B, C & D: Representative immunographs (channel-separated) of MDA-MB-231 cells immunostained for centrin-2 (green) and γ-tubulin (red) after treatment with $CoCl_2$. Where panel 1 in all three images is DAPI (Nuclei) panel 2 is γ-tubulin (red) and panel 3 is centrin-2 (green). **B:** Represents the split confocal images of dispersed centrosome aberrations. **C**: Represents the split confocal images of the clustered CA. **D**: Represents the split confocal images of structural aberration with PCM accumulation.

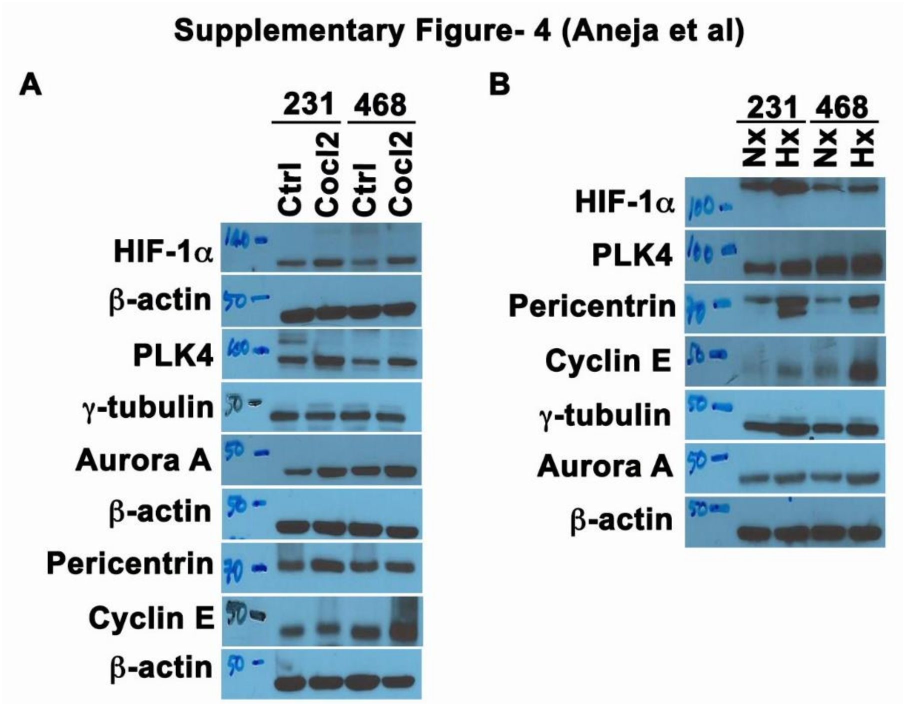

Supplementary Figure 4: Protein immunoblots. **A :** Immunoblots showing the levels of hypoxia and centrosomal markers in MDA-MB-231 and MDA-MB-468 cells treated with 100µM of $CoCl_2$ for 24 h. **B.** Immunoblots showing the levels of hypoxia and centrosomal markers in MDA-MB-231 and MDA-MB-468 cells exposed to hypoxia for 48 h.

Supplementary Figure- 5 (Aneja et al)

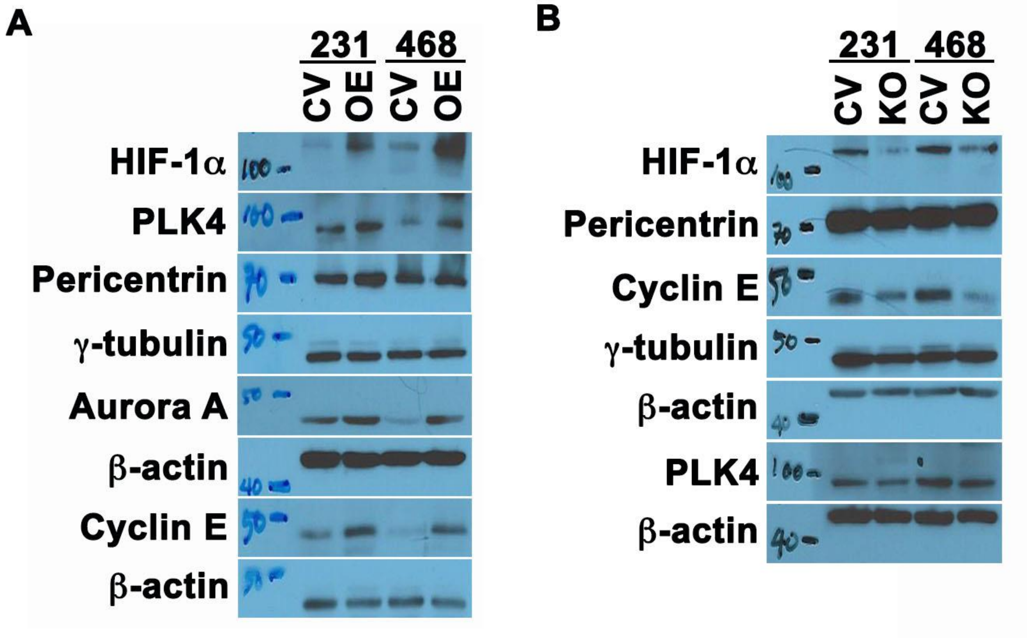

Supplementary Figure 5: Protein immunoblots. **A** Immunoblots of HIF-1α and centrosomal proteins in MDA-MB-231 and MDA-MB-468 transfected with empty vector or degradation-resistant HIF-1α. **B.** Immunoblots of HIF-1α and centrosomal proteins in MDA-MB-231 and MDA-MB-468 transfected with Cas9-sgRNA (HIF-1α) construct or control vector (pSpCas9-2A-GFP).

Supplementary Figure- 6 (Aneja et al)

A

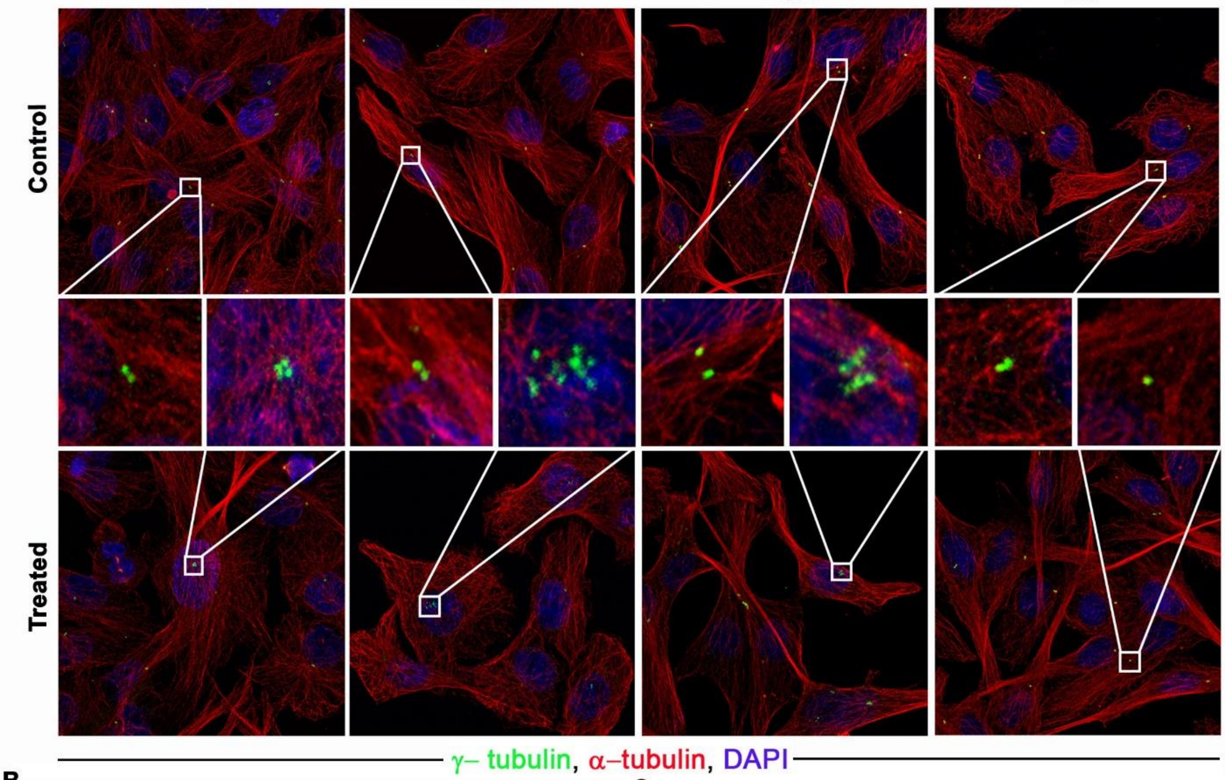

B

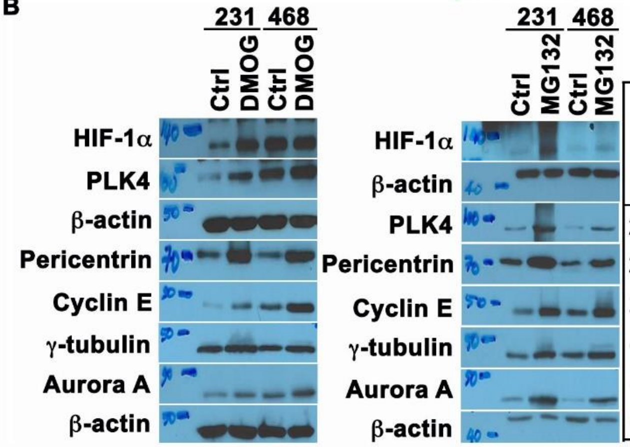

C

	Number of Cells Counted	Number of Cells with Abnormal Centrosomes	% of CA
231 DMOG	200	56	28
231 MG132	200	63	31.5
231 Control	200	38	19
468 DMOG	200	25	12.5
468 MG132	200	31	15.5
468 Control	200	17	8.5

Supplementary Figure 6: A. Representative confocal micrographs of centrosome aberrations in MDA-MB-231 cells treated with DMOG and MG132. Representative confocal micrographs of centrosome aberrations in MDA-MB-231 transfected with empty vector or degradation-resistant HIF-1α and with HIF-1α gene KO**. B.** Immunoblots showing the levels of hypoxia and centrosomal markers in cells treated with 1mM DMOG for 24 h. Immunoblots showing the levels of hypoxia and centrosomal markers in cells treated with 5µM of MG132 for 5 h**. C**. Quantitation of centrosome aberrations per microscopic examination for DMOG and MG132 treated and untreated MDA-MB-231 and MDA-MB-468 cells.

Enrichment of centrosomal gene expression in tumors with a hypoxia-high gene expression signature

We validated our in vitro findings of a correlation between CA and hypoxia in silico by probing the publicly-available Kao[1] and Jonsdottir[2] microarray datasets using Gene Set Enrichment Analysis (GSEA).[3] Essentially, our goal was to determine whether breast tumors that are enriched in hypoxia-associated transcripts also show a correlational enrichment in centrosomal transcripts. Publicly available pre-processed gene expression profiles of primary breast tumors (n=327 for the Kao dataset, GSE20685; n=94 for the Jonsdottir dataset, GSE46563) were used for GSEA. Within each dataset, patients were stratified into two groups by a hypoxia score, the reduced hypoxia metagene previously shown to have prognostic ability in multiple cancers.[4,5] As previously defined, hypoxia scores were calculated as the median expression of 26 genes that are upregulated in response to hypoxia. Scores ≤ median were categorized as “hypoxia low” and scores > median were categorized as “hypoxia high.” For the Kao dataset, Affymetrix probes with the “x_at” extension were removed unless no other probe was available (e.g., as with *ALDOA*). For the Jonsdottir dataset, Illumina probes with the “A” designator were preferentially used. When multiple probes were present, their median expression was used in score calculation. GSEA was

performed with 1000 permutations, and false discovery rate q-values<0.05 were considered statistically significant.

Using the Kao dataset, we collapsed features into gene symbols, resulting in 20,606 genes being available for GSEA using curated gene sets from Molecular Signatures Database[6] v5.0, including those from the Gene Ontology (GO) Consortium (for analysis of cellular components and biological processes) and Reactome[7] v53 (for pathway analysis), along with gene sets that we defined based on empirical evidence from the literature. We validated that the hypoxia-high group was differentially enriched in hypoxia-associated genes by performing GSEA with the full hypoxia metagene as shown in Supplementary Fig. 7 (also see Supplementary Table 1 for study details and Supplementary Table 2 for the ranked gene list; n=44 after filtering). We then performed GSEA to identify gene ontologies associated with the hypoxia-high group, which we found was significantly enriched in microtubule-organizing center and centrosome components, which were among the top-20 enriched cellular components (see Supplementary Table 1 for these and all other enriched gene ontologies). The hypoxia-high group was also enriched in cell cycle-related processes, which constituted the top-ranked gene ontology among biological processes. Cellular pathway analysis using Reactome terms identified mitosis as the third-most enriched pathway, with various other cell cycle-related pathways also significantly enriched. Cellular pathway analysis revealed an enrichment in genes associated with the recruitment of centrosome proteins and complexes. Intriguingly, the hypoxia-high group was also enriched in genes involved in the cellular pathway associated with loss of ninein-like protein (NLP), a γ-tubulin-binding protein, from mitotic centrosomes. It is known that PLK1 and NEK2 phosphorylate NLP at the onset of mitosis, resulting displacement of NLP from the centrosome, which is associated with centrosome maturation (involving the recruitment of γ-tubulin ring complexes and other pericentriolar material

components) and a concomitant increase in microtubule-nucleating capacity. PLK1 or NEK2 overexpression results in premature NLP dissociation from centrosomes and also induces CA.[8]

Although hypoxia-high breast tumors were clearly found to be enriched in centrosomal components and pathways, we wanted to more specifically test the hypothesis that they are enriched in gene ontologies related to CA per se. No high-throughput screen of CA-associated genes has been performed to inform construction of a CA gene set; nevertheless, the literature reports that CA is associated with hormone receptor-negative and node-positive breast cancer.[9] Thus, we analyzed enrichment of centrosome-associated genes (namely, experimentally identified human centrosomal proteins in the MiCroKiTS[10] database; n=540 genes) in hormone receptor-positive node-negative patients, rationalizing that this gene set has a high likelihood of representing CA. We found that 77 of these genes were enriched in hormone receptor-negative node-positive breast carcinomas. Next, we performed GSEA using these 77 genes as a gene set, which we found was significantly enriched in the hypoxia-high group, as shown in Supplementary Fig. 7B (also see Supplementary Table 3 for the ranked gene list). Many genes implicated in CA (such as *AURKA*, *CCNA2*, *CCNE2*, *CEP152*, *NEK2*, *PLK4, and STIL*) or amplified centrosome clustering (such as *KIFC1*, the top-ranked hit, along with *BIRC5* and *TACC3*) from the literature are among the enriched genes from this set. Because CA drives chromosomal instability (CIN), we wondered whether hypoxia-high cases were also enriched in CIN-associated genes. To this end, we performed GSEA with genes from the CIN25 signature, net overexpression of which has prognostic significance in various types of cancer.[11] We found this set was highly enriched in the hypoxia-high group (Supplementary Table 1). Collectively, these results suggest that hypoxic breast tumors are enriched in CA- and CIN-associated genes.

Enrichment of centrosomal gene expression in tumors with a hypoxia-high gene expression signature regardless of mitotic activity

Many CA-associated proteins do not exclusively localize to the centrosome; some also localize to the mitotic spindle. Thus, it could be argued that, rather than having a greater extent of CA, the hypoxia-high group merely has more mitotic cells than the hypoxia-low group. To test this hypothesis, we analyzed the Jonsdottir dataset, which contains gene expression profiles and mitotic activity indices for 94 breast tumor specimens from lymph node-negative patients. To begin, we validated that the hypoxia-high group was enriched in hypoxia-associated genes. We performed GSEA with the full hypoxia metagene and found significant enrichment (Supplementary Table 1), which also underscores the robustness of this 26-gene hypoxia signature across platforms and breast cancer datasets. We then performed GSEA using the 77 potentially CA-associated genes (that is, those that were enriched in the hormone receptor-negative node-positive breast carcinomas from the Kao dataset) and found significant enrichment in the Jonsdottir dataset as well (Supplementary Table 4). This is especially interesting because the Jonsdottir patients are also all node-negative, indicating this gene set captures a phenotype that is not wholly dependent on nodal status. There was substantial overlap in the potentially CA-associated genes enriched in the Jonsdottir and Kao hypoxia-high groups. Next, we did find that the hypoxia-high group was associated with a high mitotic activity index (MAI; >10 mitotic figures per 10 fields of vision) based on the Mann-Whitney test ($p=0.01$). Nonetheless, when we performed GSEA on the MAI-low group ($n=60$) using hypoxia scores as the phenotype, we still found that the hypoxia-high group was enriched in potentially CA-associated genes (Supplementary Table 5). Thus, even among tumors with relatively low mitotic activity, hypoxia-high tumors show enrichment in potentially CA-associated genes, minimizing the probability that we are merely capturing proliferation-associated genes with our gene set. Combined with our in

vitro data, these in silico data substantiate the hypothesis that hypoxia is associated with CA in patient breast tumors.

Finally, we were interested to determine whether hypoxia-associated CA, as determined by gene expression levels, predicts worse outcomes and, if so, whether its predictive ability depends on mitotic activity. To this end, we created a score based on the top ten CA-associated genes enriched in the hypoxia-high samples of the Jonsdottir dataset (from Supplementary Table 4). Specifically, we defined the hypoxia-associated CA score as the median expression of those top 10 genes. Kaplan-Meier analysis and Cox regression were performed using SPSS Statistics version 21 (IBM). For multivariate Cox regression analysis, all potential predictors were entered into the full model and then eliminated stepwise based on an $\alpha=0.10$ elimination criterion. Optimal cut points based on distant-metastasis-free survival (DMFS) were found using X-tile[12] per the highest X^2 value following dichotomization. We found that stratifying patients based on a cutpoint of 317 resulted in the CA score having the best predictive ability using the 94 node-negative breast cancer patients of the Jonsdottir dataset (p=0.020; Supplementary Fig. 6C). Univariate Cox regression revealed that a high hypoxia-associated CA score (i.e., >317) was associated with worse DMFS (HR=2.87; p=0.026), which was upheld in multivariate regression adjusting for all available potentially confounding covariates (including tumor size, Nottingham grade, estrogen and human epidermal growth factor receptor 2 statuses, and mitotic activity index). In fact, only this score remained in the final model. When hypoxia score was added to the Cox regression analysis, the effect of CA score on DMFS was more pronounced (HR=3.39, p=0.011). Only the CA score and hypoxia score remained in the final model, though the hypoxia score was no longer significant (HR=2.22, p=0.066). When the analysis was repeated without the CA score in the full model, however, the hypoxia score was a significant predictor of DMFS (HR=2.45, p=0.047), as was mitotic activity (HR=2.88, p=0.0.17), with no other variables in the final model. These results raise the tantalizing possibility that the ability of the hypoxia score to predict DMFS results from

its association with CA. Even more intriguing is the idea that hypoxia might upregulate CA to drive metastatic dissemination, an exciting avenue of future research.

Supplementary Figure- 7 (Aneja et al)

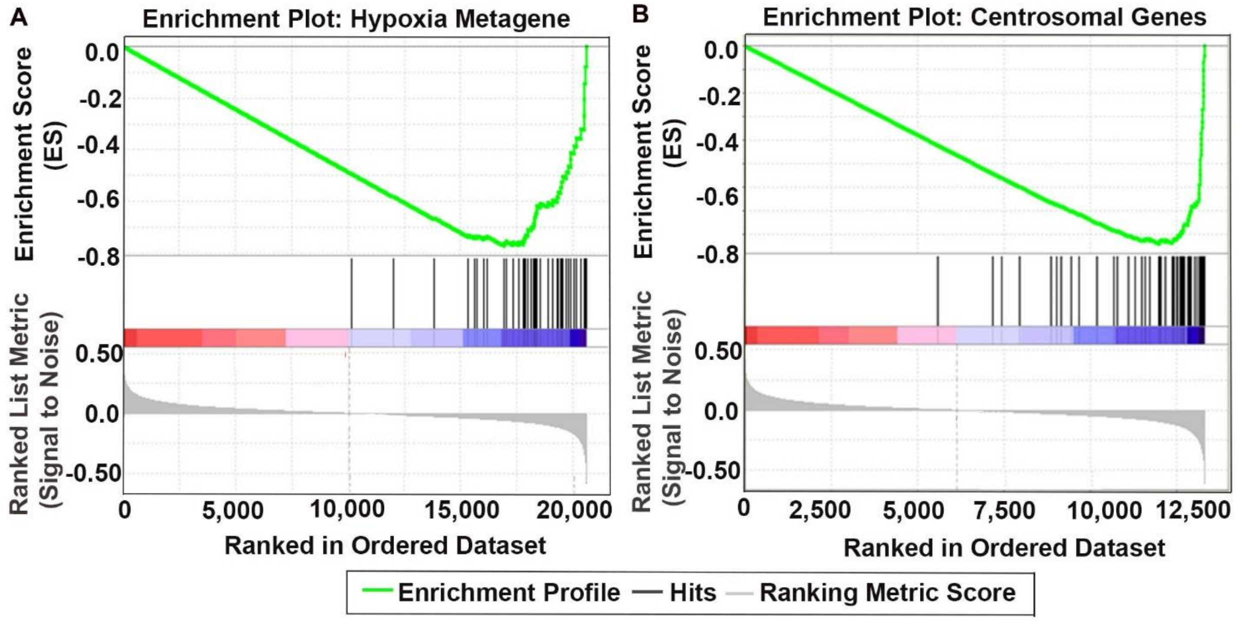

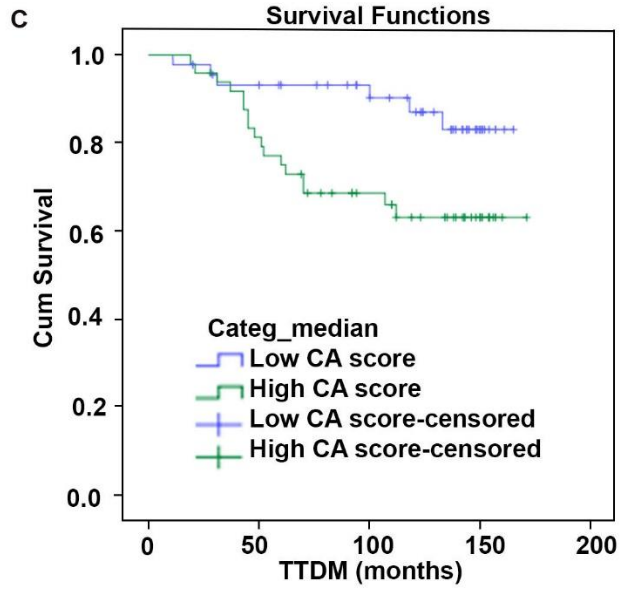

Di

CA Score	Total Cases	Distant Metastases	Censored N	Censored %
Low	45	6	39	86.7%
High	49	17	32	65.3%
Overall	94	23	71	75.5%

Dii

Mean For DMF S Time

CA Score	Estimate	Std. Error	Mean[3] 95%Confidence Interval Lower	Upper
Low	150.667	5.814	139.272	162.062
High	128.674	8.409	112.192	145.156
Overall	141.257	5.513	130.451	152.063

Supplementary Figure 7: Gene-set enrichment and Kaplan-Meier analyses based on hypoxia- and centrosome aberrations-associated genes. A. Enrichment plots of hypoxia metagene components available in the Kao dataset and **B.** genes potentially associated with centrosome aberrations (CA), with red indicating correlation with the hypoxia-low group and blue the hypoxia-high group. **C.** Plots of Kaplan-Meier product limit estimates of distant metastasis-free survival of patients in the Jonsdottir dataset stratified by hypoxia-associated CA score (low vs. high), p=0.020 by the log-rank tesst. TTDM=time to distant metastasis. **Di.** Number of distant metastases and censored cases by centrosome amplification score. **Dii.** Mean distant metastasis-free survival time by centrosome amplification score.

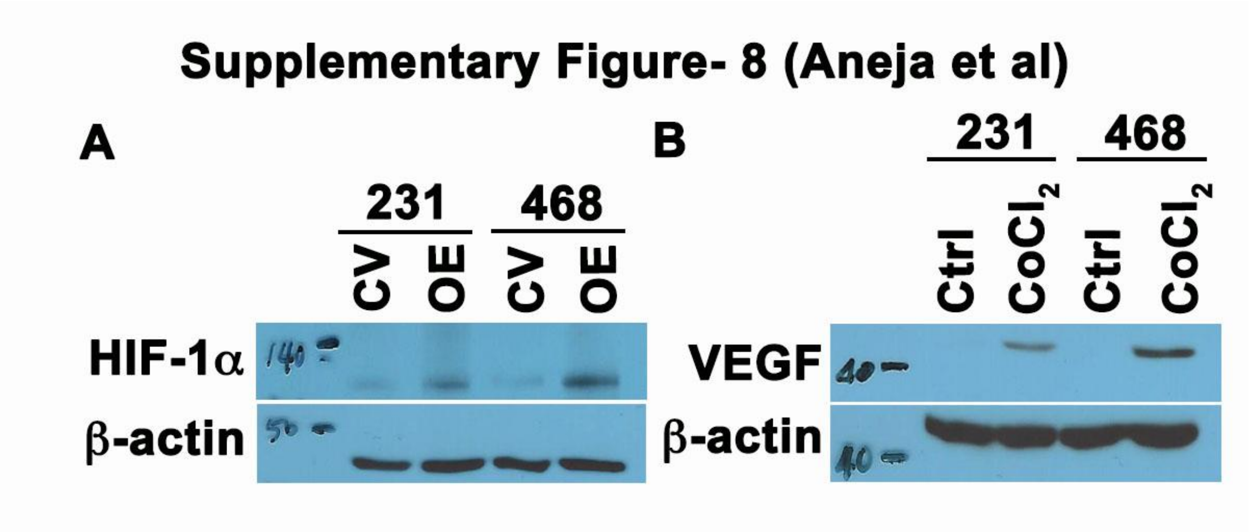

Supplementary Figure 8: Representative immunoblots **A**. Immunoblots of HIF-1α in MDA-MB-231 and MDA-MB-468 transfected with empty vector or degradation-resistant HIF-1α. **B**. Immunoblots of VEGF in MDA-MB-231 and MDA-MB-468 cells treated with 100µM of $CoCl_2$ for 24 hrs.

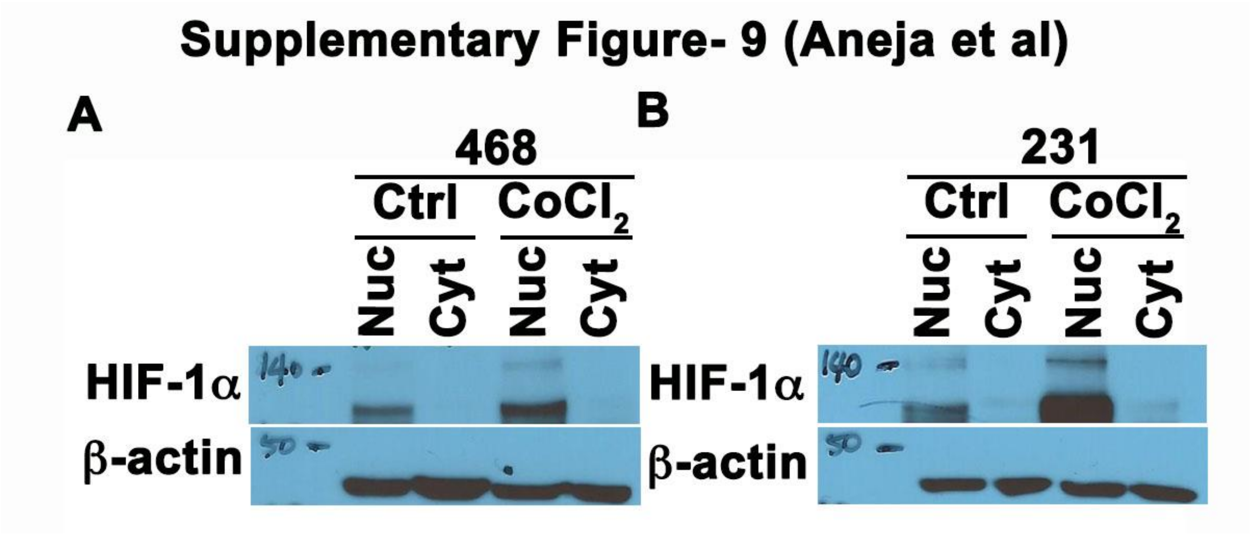

Supplementary Figure 9: Representative immunoblots showing the levels of HIF-1α in nuclear and cytoplasmic lysates of MDA-MB-231 and MDA-MB-468 cells treated with 100µM of $CoCl_2$ for 24 hrs.

Gene Set	Size after Filtering	ES	NES	NOM p-value	FDR q-value	FWER p-value	Rank at Max
**Kao - Entire Dataset**							

Hypoxia Metagene (Buffa et al., 2010)	44	-0.77	-2.04	<0.001	<0.001	<0.001	3686
Microtubule Organizing Center (GO:0005815)	57	-0.54	-2.06	<0.001	4.5E-03	0.03	1531
Centrosome (GO:0005813)	49	-0.54	-2.01	<0.001	0.01	0.05	1531
Cell cycle (GO:0007049)	285	-0.61	-2.33	<0.001	3.1E-03	0.02	2632
Cell Cycle - Mitotic (Pathway:69278)	271	-0.67	-2.29	<0.001	4.2E-03	0.01	2316
Recruitment of Mitotic Centrosome Proteins and Complexes (Pathway:380270)	54	-0.46	-1.89	0.02	0.02	0.01	2313
Loss of NLP from Mitotic Centrosomes (Pathway:380259)	47	-0.46	-1.85	0.02	0.02	0.40	2313
Centrosomal Genes Upregulated in ER/PR+ Node- Tumors	77	-0.82	-1.88	<0.001	<0.001	<0.001	2630
CIN25 Genes (Carter et al., 2006)	23	-0.89	-1.69	<0.001	<0.001	<0.001	1297
**Jonsdottir - Entire Dataset**							
Hypoxia Metagene (Buffa et al., 2010)	47	-0.81	-1.55	0.01	0.01	0.003	5057
Centrosomal Genes Upregulated in ER/PR+ Node- Tumors	77	-0.81	-1.56	4.0E-03	4.0E-03	2.0E-03	4008
**Jonsdottir - MAI-Low Only**							
Hypoxia Metagene (Buffa et al., 2010)	47	-0.75	-1.49	0.04	0.04	0.02	5163
Centrosomal Genes Upregulated in ER/PR+ Node- Tumors	77	-0.85	-1.72	<0.001	<0.001	<0.001	1813

Supplementary Table 1. Gene Set Enrichment Analyses for specified Gene Ontologies, Reactome pathways, literature-based gene sets, and self-defined gene sets. GO and Reactome IDs (for curated gene sets) and publication details (for literature-based gene sets) are given in parentheses. ES=Enrichment Score; NES=Normalized Enrichment Score; NOM=nominal; FDR=False Discovery Rate; FWER=Family-Wise Error Rate

GENE SYMBOL	RANK IN GENE LIST	RANK METRIC SCORE	RUNNING ES	CORE ENRICHMENT
ALDOA	10185	-0.001037822	-0.4951352	No
ANKRD37	12004	-0.013171702	-0.5810628	No
LRRC42	13846	-0.026962586	-0.6655041	No
MCTS1	15337	-0.04007807	-0.73039776	No
MIF	15612	-0.042822175	-0.7356349	No
CHCHD2	15748	-0.044233657	-0.7338455	No
LDHA	16016	-0.047092326	-0.7379356	No
GAPDH	16182	-0.049089462	-0.73668796	No
GPI	16921	-0.058381788	-0.76155204	Yes
SEC61G	17016	-0.059670471	-0.75485283	Yes
UTP11L	17344	-0.064875908	-0.75850195	Yes
HK2	17604	-0.069081984	-0.75804955	Yes
TUBB6	17812	-0.072616458	-0.7544006	Yes
PSMA7	17838	-0.073151112	-0.7417994	Yes
SLC25A32	17903	-0.074541941	-0.7308322	Yes
PGK1	18012	-0.076205611	-0.72169065	Yes
BNIP3	18017	-0.076291725	-0.70747495	Yes
P4HA1	18145	-0.07900098	-0.69872946	Yes
MAD2L2	18230	-0.080925092	-0.68752927	Yes
CORO1C	18285	-0.082037203	-0.67465997	Yes
ENO1	18319	-0.082770482	-0.6606309	Yes

TPI1	18340	-0.083269544	-0.64587533	Yes
SLC16A1	18401	-0.084655896	-0.63280326	Yes
YKT6	18405	-0.084711224	-0.6169486	Yes
DDIT4	18565	-0.088841394	-0.6079007	Yes
MRPL15	18878	-0.098103359	-0.60454416	Yes
MRPL13	19107	-0.106322989	-0.59554994	Yes
AK3L1	19315	-0.114598989	-0.5839712	Yes
NDRG1	19351	-0.11648801	-0.56367075	Yes
NP	19458	-0.121711895	-0.5458365	Yes
SLC2A1	19484	-0.12285845	-0.52384645	Yes
MRPS17	19534	-0.126033917	-0.50242376	Yes
ACOT7	19727	-0.138207912	-0.48565617	Yes
PFKP	19803	-0.144859448	-0.46194214	Yes
HIG2	19898	-0.152798504	-0.4376526	Yes
PSRC1	19910	-0.154252052	-0.4090519	Yes
ADM	20066	-0.17373921	-0.38377362	Yes
CA9	20175	-0.192664087	-0.35263497	Yes
CTSL2	20338	-0.228577107	-0.31733918	Yes
SHCBP1	20490	-0.299957722	-0.2680258	Yes
CDKN3	20524	-0.333389521	-0.20665893	Yes
KIF20A	20528	-0.337646633	-0.14302899	Yes

KIF4A	20545	-0.356419325	-<br>0.0764854<br>3	Yes
ANLN	20585	-0.420125276	9.73E-04	Yes

Supplementary Table 2. Rank-ordered list of filtered hypoxia metagene components with associated rank metric scores, enrichment scores (ES), and whether each gene is part of the core enriched genes (i.e., the leading-edge subset) in the hypoxia-high group of the Kao dataset.

Supplementary Table 3.

GENE SYMBOL	RANK IN GENE LIST	RANK METRIC SCORE	RUNNING ES	CORE ENRICHMENT
TRIM22	9257	0.005535	-0.45051	No
PRKAA1	10390	-0.0025	-0.50547	No
MAP1B	11819	-0.01194	-0.57415	No
GIMAP5	12039	-0.01341	-0.58383	No
FYN	12842	-0.01913	-0.62148	No
ODF2L	13067	-0.02089	-0.63085	No
RASSF5	14099	-0.02883	-0.67894	No
PRKD3	14243	-0.02992	-0.6837	No
MARCKS	14472	-0.03196	-0.69244	No
LIMK1	14658	-0.03361	-0.69897	No
SYK	14810	-0.0351	-0.70373	No
HAP1	15154	-0.03818	-0.71762	No
PIM1	16282	-0.05017	-0.76881	No
FIGN	16589	-0.05401	-0.77973	No
LIMK2	16953	-0.05886	-0.79306	No
BRSK1	17048	-0.06002	-0.79321	No
PRKCQ	17094	-0.06075	-0.79091	No
SMURF2	17543	-0.06803	-0.80771	No
SOCS1	17796	-0.07242	-0.81464	No
NEK6	17977	-0.07552	-0.81783	Yes
CRYAB	18091	-0.07784	-0.81758	Yes
RUNX3	18196	-0.08005	-0.81673	Yes

SPHK1	18224	-0.0808	-0.81208	Yes
SASS6	18240	-0.08116	-0.80682	Yes
SLC16A1	18401	-0.08466	-0.80836	Yes
GNAI1	18457	-0.08613	-0.80467	Yes
TSKS	18806	-0.09576	-0.81455	Yes
WASF1	18833	-0.0967	-0.80867	Yes
PLEKHG6	19038	-0.10343	-0.81097	Yes
PDE7A	19279	-0.11301	-0.81431	Yes
LCK	19344	-0.11619	-0.80885	Yes
NDRG1	19351	-0.11649	-0.80054	Yes
CCNA1	19406	-0.1191	-0.79437	Yes
CENPJ	19444	-0.12114	-0.78722	Yes
LMO4	19518	-0.12512	-0.78154	Yes
PSMD3	19615	-0.13063	-0.77656	Yes
VAC14	19642	-0.13237	-0.76805	Yes
CDC25B	19705	-0.13691	-0.76096	Yes
CHEK2	19769	-0.14153	-0.75357	Yes
RANBP1	19788	-0.14291	-0.74389	Yes
GPSM2	19923	-0.15544	-0.73894	Yes
CKAP2	19951	-0.15892	-0.72852	Yes
CEP152	19990	-0.16318	-0.71831	Yes
MCM5	19996	-0.16407	-0.70644	Yes
GMNN	20040	-0.16928	-0.69603	Yes
EIF4EBP1	20052	-0.17204	-0.68385	Yes
TACC3	20230	-0.20252	-0.67752	Yes
FANCA	20250	-0.20734	-0.66313	Yes
BRCA2	20291	-0.21723	-0.64903	Yes
SGOL1	20325	-0.22475	-0.63403	Yes
STIL	20384	-0.24633	-0.61866	Yes
ESPL1	20406	-0.25368	-0.60095	Yes
CHEK1	20417	-0.2561	-0.58252	Yes
TROAP	20421	-0.25745	-0.56365	Yes
CCNB1	20430	-0.26098	-0.54476	Yes
PTTG1	20441	-0.26845	-0.52542	Yes
CRABP1	20449	-0.27154	-0.5057	Yes
CCNA2	20464	-0.28031	-0.48567	Yes
KIF15	20467	-0.28398	-0.4648	Yes
MAD2L1	20468	-0.28479	-0.44376	Yes
CENPF	20471	-0.28903	-0.42251	Yes
AURKA	20480	-0.29411	-0.40117	Yes
KIF11	20487	-0.29864	-0.3794	Yes
HMMR	20503	-0.31248	-0.35705	Yes
CDC25C	20510	-0.32075	-0.33365	Yes

CCNE2	20511	-0.32077	-0.30996	Yes
NEK2	20517	-0.32682	-0.28606	Yes
CDKN3	20524	-0.33339	-0.26172	Yes
KIF14	20548	-0.35903	-0.23632	Yes
PLK1	20550	-0.35933	-0.20983	Yes
KIF18A	20556	-0.37178	-0.18261	Yes
ASPM	20557	-0.37248	-0.1551	Yes
TTK	20560	-0.37723	-0.12733	Yes
CEP55	20561	-0.37875	-0.09935	Yes
BIRC5	20577	-0.40346	-0.07028	Yes
CDC20	20586	-0.42014	-0.03964	Yes
KIFC1	20603	-0.54844	9.75E-05	Yes

Supplementary Table 3. Rank-ordered list of potential centrosome aberrations-associated genes with associated rank metric scores, enrichment scores (ES), and whether each gene is part of the core enriched genes (i.e., the leading-edge subset) in the hypoxia-high group of the Kao dataset.

GENE SYMBOL	RANK IN GENE LIST	RANK METRIC SCORE	RUNNING ES	CORE ENRICHMENT
MAP1B	9927	0.027879	-0.2694731	No
GIMAP5	11620	0.018845	-0.3146127	No
NEK6	14304	0.006694	-0.3874912	No
TSKS	20675	-0.02045	-0.5602645	No
LCK	21643	-0.0252	-0.585253	No
GNAI1	21692	-0.02545	-0.5851359	No
MARCKS	25609	-0.04594	-0.6894773	No
SYK	25673	-0.04634	-0.6885981	No
WASF1	25818	-0.04722	-0.689881	No
FIGN	26288	-0.05058	-0.6998489	No
SMURF2	26579	-0.05269	-0.704811	No

CCNB1	26876	-0.05483	-0.7098172	No
TRIM22	28230	-0.06621	-0.7430442	No
SLC16A1	28325	-0.06714	-0.7418446	No
BRCA2	29320	-0.07824	-0.764595	No
RASSF5	29744	-0.08353	-0.7714585	No
PRKAA1	30331	-0.0924	-0.7822752	No
HAP1	30451	-0.09399	-0.780252	No
ODF2L	30904	-0.10177	-0.7868843	No
FYN	31621	-0.1156	-0.7999487	No
PRKD3	31760	-0.11896	-0.7970437	No
PSMD3	32154	-0.12857	-0.8005617	Yes
SOCS1	32256	-0.13107	-0.7959673	Yes
CRABP1	32696	-0.14402	-0.7998746	Yes
CCNA1	32921	-0.15105	-0.7975177	Yes
SASS6	33107	-0.15692	-0.7937663	Yes
BRSK1	33320	-0.16365	-0.7903748	Yes
CHEK2	33524	-0.17026	-0.7863668	Yes
RUNX3	34028	-0.19051	-0.7894138	Yes
LIMK1	34450	-0.20953	-0.789155	Yes
KIF14	34790	-0.22531	-0.7857721	Yes
SPHK1	34813	-0.22655	-0.7736645	Yes
TACC3	34830	-0.22751	-0.7613397	Yes
PRKCQ	34832	-0.22752	-0.7486046	Yes
ESPL1	34847	-0.22799	-0.7361981	Yes
FANCA	34935	-0.23268	-0.7255213	Yes
CDC25C	34988	-0.23525	-0.7137451	Yes
VAC14	35284	-0.25222	-0.7076517	Yes
HMMR	35293	-0.25279	-0.6936903	Yes
PLEKHG6	35543	-0.27288	-0.6851819	Yes
CDC25B	35587	-0.27725	-0.6708041	Yes
PLK1	35601	-0.27839	-0.655543	Yes

EIF4EBP1	35641	-0.28117	-0.6408358	Yes
CRYAB	35712	-0.28594	-0.6267073	Yes
MCM5	35760	-0.29006	-0.61172	Yes
CKAP2	35792	-0.29443	-0.5960506	Yes
ASPM	35799	-0.29504	-0.5796646	Yes
KIF11	35823	-0.29804	-0.5635741	Yes
CCNE2	35873	-0.30251	-0.5479429	Yes
GMNN	35987	-0.31268	-0.5334887	Yes
CENPJ	35995	-0.31324	-0.5161092	Yes
NEK2	36018	-0.31532	-0.4990226	Yes
CEP55	36028	-0.31635	-0.4815231	Yes
CDKN3	36053	-0.31868	-0.4643022	Yes
PIM1	36092	-0.32313	-0.4472143	Yes
BIRC5	36106	-0.32495	-0.4293416	Yes
KIF18A	36150	-0.3296	-0.4120271	Yes
MAD2L1	36155	-0.33	-0.3936251	Yes
CENPF	36214	-0.33744	-0.3762802	Yes
PTTG1	36249	-0.34282	-0.3579785	Yes
TROAP	36260	-0.3446	-0.3389218	Yes
KIF15	36303	-0.35197	-0.3203251	Yes
CEP152	36317	-0.35415	-0.3008146	Yes
SGOL1	36319	-0.35452	-0.2809555	Yes
TTK	36354	-0.3624	-0.2615553	Yes
CDC20	36396	-0.36887	-0.2419837	Yes

CCNA2	36406	-0.37126	-0.221404	Yes
KIFC1	36418	-0.37251	-0.2008091	Yes
LIMK2	36451	-0.37996	-0.1803693	Yes
AURKA	36460	-0.38249	-0.1591323	Yes
PDE7A	36488	-0.38882	-0.1380591	Yes
GPSM2	36559	-0.4058	-0.1172072	Yes
NDRG1	36567	-0.40833	-0.0944937	Yes
RANBP1	36571	-0.40905	-0.0716305	Yes
LMO4	36595	-0.41683	-0.0488768	Yes
STIL	36597	-0.41794	-0.02546	Yes
CHEK1	36684	-0.50451	4.92E-04	Yes

Supplementary Table 4. Rank-ordered list of potential centrosome aberrations-associated genes with associated rank metric scores, enrichment scores (ES), and whether each gene is part of the core enriched genes (i.e., the leading-edge subset) in the hypoxia-high group of the entire Jonsdottir dataset.

GENE SYMBOL	RANK IN GENE LIST	RANK METRIC SCORE	RUNNING ES	CORE ENRICHMENT
CRABP1	1247	0.196209	-0.022491	No
SYK	4586	0.083097	-0.108734	No
MAP1B	4941	0.077617	-0.113828	No
GNAI1	6693	0.05668	-0.158298	No
PRKCQ	7260	0.051505	-0.170718	No
CCNA1	11454	0.025851	-0.283677	No
GIMAP5	12204	0.022238	-0.302817	No

LIMK1	13681	0.015818	-0.342184	No
TSKS	14283	0.013139	-0.35782	No
HAP1	14837	0.010819	-0.372281	No
NEK6	15431	0.008267	-0.387985	No
LCK	16809	0.002983	-0.425406	No
RUNX3	18761	-0.004223	-0.478425	No
WASF1	19448	-0.006835	-0.496752	No
SMURF2	20802	-0.012268	-0.532971	No
SOCS1	21207	-0.014023	-0.543175	No
CRYAB	23902	-0.025877	-0.615206	No
PRKD3	24183	-0.027193	-0.621249	No
SLC16A1	24424	-0.028282	-0.626136	No
FANCA	24474	-0.028508	-0.625795	No
TRIM22	26151	-0.036923	-0.66938	No
ODF2L	26970	-0.041393	-0.689276	No
LMO4	27255	-0.042918	-0.694502	No
VAC14	28281	-0.049818	-0.719554	No
FIGN	28767	-0.053333	-0.729655	No
MARCKS	28812	-0.053722	-0.727692	No
EIF4EBP1	30948	-0.074622	-0.781589	No
CHEK2	31454	-0.081308	-0.790588	No
RASSF5	32122	-0.091782	-0.803394	No
FYN	32229	-0.093444	-0.800785	No
PRKAA1	33495	-0.122161	-0.828128	No
SPHK1	33904	-0.13265	-0.831455	No
BRCA2	34464	-0.152763	-0.837721	No
PIM1	34891	-0.169697	-0.839357	Yes
BRSK1	35120	-0.181113	-0.834915	Yes
LIMK2	35200	-0.184675	-0.826196	Yes
PDE7A	35419	-0.198272	-0.82047	Yes
PLEKHG6	35542	-0.20546	-0.811701	Yes
PSMD3	35718	-0.219175	-0.80357	Yes
GMNN	35762	-0.222346	-0.791649	Yes
NDRG1	35901	-0.235135	-0.781568	Yes
RANBP1	35918	-0.236222	-0.768093	Yes
MCM5	35984	-0.242655	-0.755576	Yes
PLK1	36105	-0.258397	-0.743634	Yes
SASS6	36241	-0.277588	-0.730971	Yes
CEP152	36386	-0.312943	-0.716472	Yes
CENPF	36392	-0.313983	-0.698116	Yes

CDC25B	36411	-0.319583	-0.679785	Yes
STIL	36430	-0.324553	-0.661162	Yes
ASPM	36438	-0.326208	-0.642141	Yes
AURKA	36476	-0.341741	-0.623024	Yes
KIF14	36482	-0.342514	-0.602988	Yes
BIRC5	36494	-0.345894	-0.582916	Yes
CCNB1	36500	-0.348213	-0.562544	Yes
KIF18A	36520	-0.356315	-0.542078	Yes
TACC3	36528	-0.35818	-0.521174	Yes
HMMR	36541	-0.363636	-0.500085	Yes
CKAP2	36568	-0.374923	-0.478713	Yes
MAD2L1	36570	-0.37601	-0.456595	Yes
SGOL1	36580	-0.38294	-0.434287	Yes
CCNE2	36581	-0.383755	-0.411685	Yes
KIFC1	36583	-0.383929	-0.389101	Yes
CCNA2	36587	-0.38714	-0.366382	Yes
GPSM2	36588	-0.388246	-0.343516	Yes
CDKN3	36616	-0.40076	-0.32065	Yes
CDC20	36621	-0.406762	-0.296802	Yes
ESPL1	36626	-0.410511	-0.272734	Yes
CHEK1	36627	-0.412625	-0.248432	Yes
TTK	36633	-0.414843	-0.224136	Yes
CEP55	36647	-0.434241	-0.198916	Yes
TROAP	36665	-0.460532	-0.172257	Yes
KIF11	36671	-0.467194	-0.144878	Yes
CDC25C	36678	-0.47623	-0.116993	Yes
KIF15	36679	-0.477443	-0.088874	Yes
NEK2	36683	-0.486579	-0.060298	Yes
PTTG1	36691	-0.501135	-0.030975	Yes
CENPJ	36699	-0.530558	8.18E-05	Yes

Supplementary Table 5. Rank-ordered list of potential centrosome aberrations-associated genes with associated rank metric scores, enrichment scores (ES), and whether each gene is part of the core enriched genes (i.e., the leading-edge subset) in the hypoxia-high group of the mitosis-activity-index-low cases of the Jonsdottir dataset.

Experiment		HIF-1α	PlK4	Cyclin-E	γ-tubulin	Pericentrin	Aurora-A
Fig.4B- Hypoxia treatment	231 NX	0.867309	1.088736	0.764054	0.940908	0.084236	0.941012
	231 HY	1.427749	1.458914	1.151796	1.314168	0.285311	1.181304
	468 NX	0.882063	1.088638	0.54138	0.12408	0.114685	0.812575
	468 HY	1.076276	1.74581	0.979746	1.0245	0.606658	1.044164
Fig.5B- Hypoxia induced by Cocl2 treatment	231 Ctrl	0.218511	0.832513	0.294373	0.592241	0.069429	0.832411
	231 Trt	1.239352	1.590948	2.493794	0.804104	1.931564	1.231247
	468 Ctrl	0.28707	0.826657	1.873522	0.703674	0.29214	0.832097
	468 Trt	2.680947	1.305258	3.325025	1.267246	3.23625	1.262659
Fig.5C- HIF-1α OE	231 CV	0.747236	0.670742	0.85962	1.847637	1.476639	0.894344
	231 OE	1.72515	1.118433	1.09351	2.473519	2.492973	2.605576
	468 CV	0.621214	0.971311	0.088223	2.082607	3.132659	1.645612
	468 OE	3.075068	3.068379	1.171741	4.687624	4.226323	3.500129
Fig.5C- HIF-1α KO	231 CV	0.663849	0.968604	0.937503	1.094114	0.874438	NA
	231 KO	0.219866	0.535841	0.535841	0.733562	0.799145	NA
	468 CV	0.962027	0.937549	0.937549	1.094106	0.825789	NA
	468 KO	0.392876	0.298447	0.298447	1.01252	0.717745	NA
Fig 6- Patient tumor samples	Normal	0.562684	0.192416	NA	0.065889	0.07082	NA
	TNBC	1.393741	1.526139	NA	1.41868	1.661001	NA
	Normal	0.834917	0.182812	NA	0.016321	0.182492	NA
	Non-TNBC	3.165156	3.103829	NA	1.419274	1.980062	NA
Supplementary Fig.6 B-DMOG treatment	231 Ctrl	0.070699	0.129414	0.06178	0.102232	0.241046	0.244206
	231 Trt	0.730699	0.639978	0.959441	0.192495	0.931273	0.882121
	468 Ctrl	0.201942	0.336708	0.109773	0.14167	0.226087	0.400704
	468 Trt	0.766199	0.685804	0.788588	0.166921	0.76518	0.888424
	231 Ctrl	0.123484	0.080159	0.091875	0.129839	0.07909	0.117454

Supplementary Fig. 6 C- MG132 treatment	231 Trt	0.893662	0.491866	0.797712	0.908607	0.83877	0.691196
	468 Ctrl	0.10509	0.089896	0.165372	0.341801	0.161174	0.1077
	468 Trt	0.537223	0.427091	0.534221	0.908613	0.923808	0.264049

Supplementary Table 6: Densitometry values relative to loading control β-actin calculated using Image-J for immunoblot assays provided in main manuscript and supplementary data.

References

1 Kao, K.-J., Chang, K.-M., Hsu, H.-C. & Huang, A. T. Correlation of microarray-based breast cancer molecular subtypes and clinical outcomes: implications for treatment optimization. *BMC cancer* **11**, 143-143, doi:10.1186/1471-2407-11-143 (2011).

2 Jonsdottir, K. *et al.* Prognostic value of gene signatures and proliferation in lymph-node-negative breast cancer. *PLoS One* **9**, e90642, doi:10.1371/journal.pone.0090642 (2014).

3 Subramanian, A. *et al.* Gene set enrichment analysis: a knowledge-based approach for interpreting genome-wide expression profiles. *Proceedings of the National Academy of Sciences of the United States of America* **102**, 15545-15550, doi:10.1073/pnas.0506580102 (2005).

4 Eustace, A. *et al.* A 26-gene hypoxia signature predicts benefit from hypoxia-modifying therapy in laryngeal cancer but not bladder cancer. *Clinical cancer research : an official journal of the American Association for Cancer Research* **19**, 4879-4888, doi:10.1158/1078-0432.ccr-13-0542 (2013).

5 Buffa, F. M., Harris, A. L., West, C. M. & Miller, C. J. Large meta-analysis of multiple cancers reveals a common, compact and highly prognostic hypoxia metagene. *Br J Cancer* **102**, 428-435, doi:10.1038/sj.bjc.6605450 (2010).

6 Liberzon, A. *et al.* Molecular signatures database (MSigDB) 3.0. *Bioinformatics* **27**, 1739-1740, doi:10.1093/bioinformatics/btr260 (2011).

7 Matthews, L. *et al.* Reactome knowledgebase of human biological pathways and processes. *Nucleic acids research* **37**, D619-D622, doi:10.1093/nar/gkn863 (2009).

8 Rapley , J. *et al.* Coordinate Regulation of the Mother Centriole Component Nlp by Nek2 and Plk1 Protein Kinases. *Molecular and Cellular Biology* **25**, 1309-1324, doi:10.1128/mcb.25.4.1309-1324.2005 (2005).

9 Schneeweiss, A. *et al.* Centrosomal aberrations in primary invasive breast cancer are associated with nodal status and hormone receptor expression. *International journal of cancer. Journal international du cancer* **107**, 346-352, doi:10.1002/ijc.11408 (2003).

10 Huang, Z. *et al.* MiCroKiTS 4.0: a database of midbody, centrosome, kinetochore, telomere and spindle. *Nucleic acids research* **43**, D328-D334, doi:10.1093/nar/gku1125 (2015).

11 Carter, S. L., Eklund, A. C., Kohane, I. S., Harris, L. N. & Szallasi, Z. A signature of chromosomal instability inferred from gene expression profiles predicts clinical outcome in multiple human cancers. *Nat Genet* **38**, 1043-1048, doi:http://www.nature.com/ng/journal/v38/n9/suppinfo/ng1861_S1.html (2006).

12 Camp, R. L., Dolled-Filhart, M. & Rimm, D. L. X-Tile: A New Bio-Informatics Tool for Biomarker Assessment and Outcome-Based Cut-Point Optimization. *Clinical Cancer Research* **10**, 7252-7259, doi:10.1158/1078-0432.ccr-04-0713 (2004).
